# Q‐GEM: Quantum Chemistry Knowledge Fusion Geometry‐Enhanced Molecular Representation for Property Prediction

**DOI:** 10.1002/advs.202504867

**Published:** 2025-06-20

**Authors:** Zhijiang Yang, Liangliang Wang, Tengxin Huang, Yunfan Wang, Mingchi Gao, Tingjun Hou, Junjie Ding, Junhua Xiao

**Affiliations:** ^1^ State Key Laboratory of Chemistry for NBC Hazards Protection Beijing 102205 P. R. China; ^2^ Innovation Institute for Artificial Intelligence in Medicine of Zhejiang University College of Pharmaceutical Sciences Zhejiang University Hangzhou 310058 P. R. China

**Keywords:** graph neural network, machine learning, molecular representation, property prediction

## Abstract

Recently, various self‐supervised learning (SSL) methods based on 3D graph neural networks (GNNs) have been developed to comprehensively represent the structural information of molecules in 3D space; this is essential for discovering new drugs. However, existing methods fail to comprehensively characterize the 3D structures of molecules and neglect the electronic structural information that significantly influences key properties such as molecular reactivity, strong electrostatic interactions, and chemical adsorption. Therefore, here, a novel molecular representation learning method is constructed, Q‐GEM, incorporating quantum and geometric structural information enhancement, based on the quantum chemical property database QuanDB and SSL methods. Q‐GEM comprises a GNN embedded with the molecular electronic and complete 3D geometrical structural information as well as several well‐designed multiscale SSL tasks, achieving superior absolute molecular conformation prediction and conformational discrimination. The Q‐GEM achieved state‐of‐the‐art performance in 12 out of 13 prediction tasks on the MoleculeNet dataset, with an average performance improvement of 3.3% and 2.0% for classification and regression prediction tasks, respectively. Moreover, an average performance improvement of 5.2% is achieved in three localized quantum chemical properties, fully demonstrating the excellent performance of Q‐GEM in distinguishing molecular electronic structures. The Q‐GEM represents a novel, powerful breakthrough for accurate molecular property[Supplementary-material advs70375-supitem-0001]prediction.

## Graphical abstract

This work introduces E‐GeoGNN, a graph neural network framework that systematically encodes molecular structural information through three hierarchical correlation graphs: atom‐bond graph, bond‐angle graph, and angle‐dihedral graph. By incorporating quantum and geometric dual‐scale self‐supervised pretraining, Q‐GEM is proposed, a novel molecular representation learning algorithm. Q‐GEM exhibits remarkable discriminative capability in distinguishing absolute molecular configurations and subtle conformational differences.

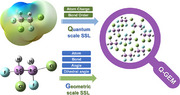

## 1 Introduction

Determination of molecular properties is crucial in drug discovery, which is related to the bioactivity and pharmacokinetic properties of the drug, such as absorption, distribution, metabolism, and excretion. In recent years, machine learning, particularly deep‐learning methods, has achieved notable results in the field of drug molecular property prediction, and the corresponding algorithms have been extended to other molecular design tasks,^[^ ^1^, ^2^, ^3^, ^4^, ^5^, ^6^ ^]^ including chemical reaction prediction,^[^ ^7^ ^]^ molecule generation,^[^ ^8^, ^9^, ^10^ ^]^ and conformer generation.^[^ ^11^, ^12^ ^]^ Furthermore, advanced machine learning techniques also exhibit remarkable potential for anomaly detection and fault diagnosis in physico‐chemical industrial processes.^[^ ^13^, ^14^, ^15^ ^]^ Notably, for downstream tasks of molecular design, the first and fundamental goal is to obtain the molecular representation. Comprehensive representation is the core factor in reducing modeling bias for various molecular discovery tasks, as it directly determines whether the mapping from molecular chemical space to functional space can be constructed efficiently.^[^ ^16^ ^]^ Several molecular representation methods have been developed, e.g., the use of a set of specific substructures to map the molecule to a fixed dimensional vector^[^ ^17^ ^]^ and sequence models to encode the molecule directly through simplified molecular input line entry system (SMILES) strings.^[^ ^18^, ^19^, ^20^ ^]^ Further, because graphs intuitively reflect molecular structures, researchers have developed many molecular representation models based on graph neural networks (GNNs), which have been applied to various tasks, such as molecular property prediction, yielding acceptable results.^[^ ^21^, ^22^, ^23^ ^]^

However, the traditional graph representation with atoms as nodes and chemical bonds as edges only learns the 2D topology of a molecule and cannot capture the 3D geometric structure information, which is more critical to molecular properties. Therefore, with the deepening understanding of molecular structure‐property relationships and the development of geometric deep‐learning (GDL), some recent studies have attempted to introduce 3D geometric structure information into traditional molecular graphs to construct 3D GNNs with invariant or equivariant nature;^[^ ^24^ ^]^ these approaches aim to enhance their representation capabilities while adhering to fundamental chemical intuitions regarding molecular structures.^[^ ^25^, ^26^, ^27^, ^28^, ^29^, ^30^ ^]^ Although the reported 3D GNNs have the ability to learn molecular 3D geometrical structure information to a certain extent, there is still a lack of a widely recognized GNN representation model for the complete extraction of such information. The absolute conformations of molecules are determined by bond lengths, bond angles, and dihedral angles; therefore, a representation model that captures the complete molecular 3D geometrical structure information could distinguish between the conformational and (or) conformational differences, which will effectively enhance the performance of the model in various downstream molecular design tasks.

Apart from the insufficient representation of 3D geometrical structures, another constraint to the performance of molecular property prediction is the limited availability of experimental data. Owing to the high cost of wet experiments, obtaining substantial experimental data to support the application of data‐driven deep‐learning methods is usually challenging. To address this limitation, inspired by the pre‐training and fine‐tuning strategies successfully applied in natural language processing^[^ ^18^, ^20^ ^]^ and computer vision,^[^ ^31^, ^32^ ^]^ the application of self‐supervised learning (SSL) to molecular property prediction tasks has garnered significant attention.^[^ ^33^, ^34^, ^35^, ^36^, ^37^ ^]^ Pre‐training on robust unlabeled data for a predefined task is first performed to learn specific chemical information. After pre‐training, only a few labeled samples are required to fit the target endpoint. With this strategy, the deep‐learning model can learn the chemical information of a molecule from large unlabeled data, making the molecular representation rational, effective, and generalizable and improving the performance of the model on the FEW‐, ZERO‐shot.^[^ ^38^ ^]^ Similarly, enhancing the performance of molecular property prediction models by introducing chemical knowledge has become consensus;^[^ ^19^, ^39^ ^]^ however, the current SSL task for molecular representation focuses more on 3D geometrical structural information and ignores the microscopic quantum chemical state, i.e., electronic structural information, which has a more significant impact on some molecular properties. Thus, the reasonable use of quantum chemical information may help enhance the performance of property‐prediction models.

In this work, we proposed a quantum fusion geometry‐enhanced molecular representation learning method termed Q‐GEM. First, to achieve a comprehensive representation of the 3D geometric structure of molecules, inspired by GEM,^[^ ^28^ ^]^ we have constructed an enhanced geometry‐based GNN architecture (E‐GeoGNN), which integrates information on atoms, chemical bonds, bond angles, and dihedral angles. The input to this architecture comprises three graphs: the atom‐bond, bond‐angle and angle‐dihedral graph. The first graph represents a conventional molecular graph, where atoms serve as nodes, and the chemical bonds connecting them act as edges. In the second graph, chemical bonds are considered as nodes, with the angles formed by two chemical bonds representing edges. Finally, in the third graph, bond angles are treated as nodes, and the dihedral angles formed by the planes containing two bond angles are considered as edges. Next, the E‐GeoGNN was pre‐trained using Zinc20^[^ ^40^ ^]^ and QuanDB^[^ ^41^ ^]^ datasets to enable the model to learn the complete 3D geometric structure on a large‐scale rough conformation, as well as the information of the electronic structure on a small‐scale stable conformation, respectively. To validate the performance of the model, we compare it with other models on the standard dataset MoleculeNet,^[^ ^42^ ^]^ and the experimental results show that Q‐GEM achieves state‐of‐the‐art (SOTA) in 12 out of the 13 benchmark tasks. Furthermore, in the local quantum chemical property prediction task, the prediction error of Q‐GEM is reduced by 5.2% on average relative to other methods. The primary contributions of this work are:

- The development of a novel GNN that explicitly integrates atomic, chemical bond, and bond angle with dihedral angle information to completely encode the molecular absolute conformation.
- The design of two levels of SSL tasks, 3D geometry and electronic structure, enabling the model to acquire accurate 3D geometry and electronic structure information of the molecules.
- A detailed discussion on the influence of dihedral angles and electronic structure information, along with the input conformations, on the molecular representation and property prediction capabilities.

## 2 The Framework of Q‐GEM

Inspired by GEM, we developed Q‐GEM, a learning framework for molecular representation that fuses molecular 3D geometry with electronic structure features. The framework comprises two parts: an enhanced geometry‐based GNN architecture (E‐GeoGNN) and a multiscale SSL task. E‐GeoGNN encodes global geometric information, including distances, bond angles, and dihedral angles, by facilitating information transfer across three graphs: atom‐bond, bond‐angle, and angle‐dihedral graph. Furthermore, through pre‐training with SSL tasks at the 3D geometric structure and electronic structure levels, the model is enabled to learn the absolute configuration and electronic structure of the molecules.

### 2.1 E‐GeoGNN

**Figure 1:**
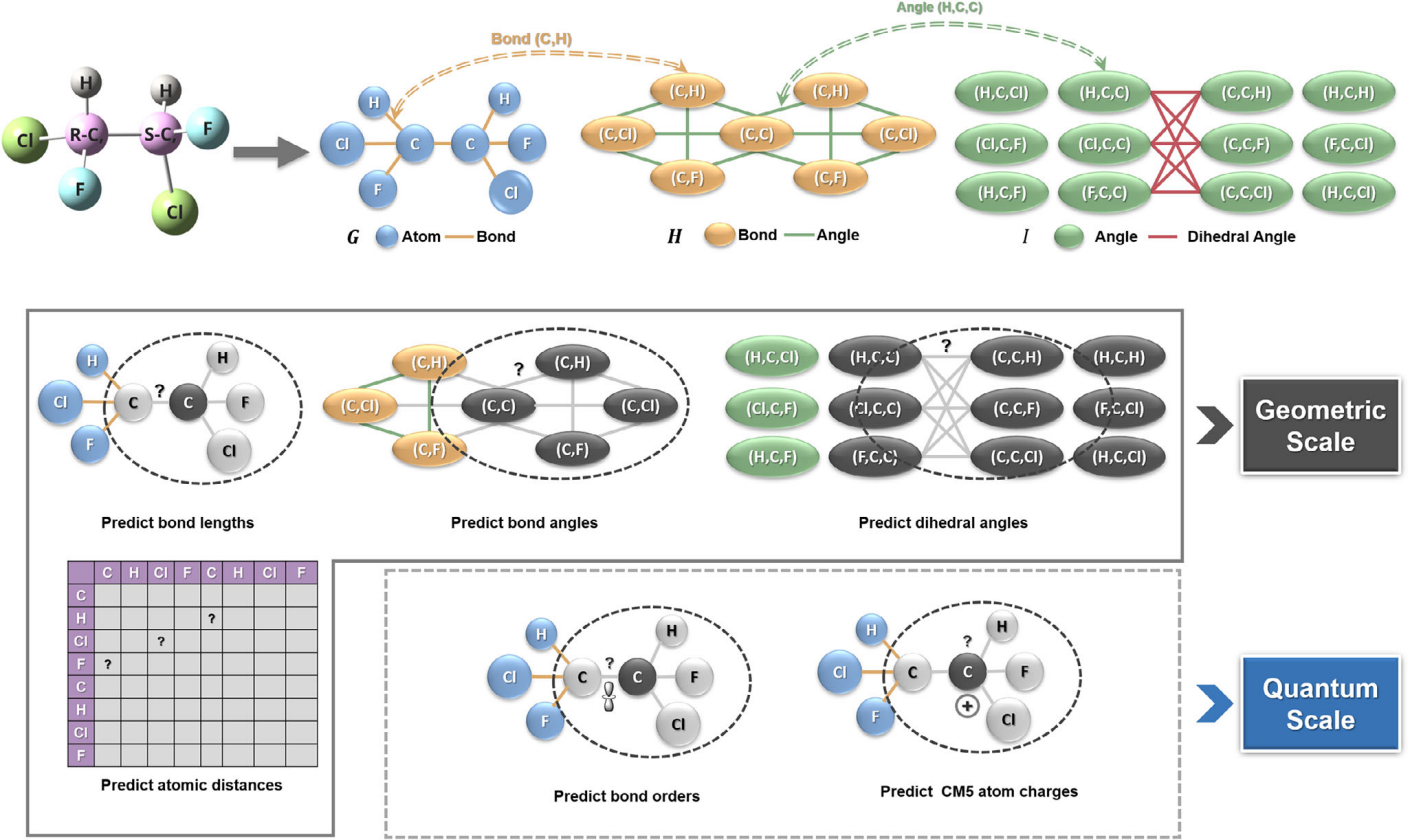
Three Input Graphs of E‐GeoGNN. In the atom‐bond graph *G*, the chemical bonds are regarded as the edges connecting the atoms. In the bond‐angle graph *H*, the bond angles are regarded as edges; a bond angle connects two chemical bonds and three atoms. In the bond angle‐dihedral graph *I*, the bond angles are regarded as nodes, while the dihedral angles within the plane of the bond angles are regarded as edges. The double‐dash arcs indicate the correspondence between the elements in the two graphs.

Distinguished from other GNN models, the input of E‐GeoGNN comprises three graphs: atom‐chemical bonding graph *G*  = (*V*,  *E*) , bond‐angle graph *H*  = (*E*,  *A*)  and bonding angle‐dihedral graph *I*  = (*A*, *D*)  (**Figure** **1**a). Where *V* is the set of atoms, *E* is the set of chemical bonds, *A* is the set of bond angles, and *D* is the set of dihedral angles. In graph *G*, atoms u∈V are considered as the graph nodes, and chemical bond (u,v)∈E connecting atoms *u* and *v* is considered as the graph edge, whereas the nodes of graph *H* are the chemical bonds (u,v)∈E, and bond angle (u,v,w)∈A connecting the bond (*u*, *v*) and (*v*, *w*) compositions is considered the graph edge. Finally, for graph *I*, the bond angle (u,v,w)∈A is considered as the graph node, and the dihedral angle (u,v,w,x)∈D formed by the bond angles (*u*, *v*, *w*) and (*v*, *w*, *x*) is considered as the edge. In addition, we used **x**_*u*_ to denote the initial feature of atom *u*, **x**_*uv*_ to denote the feature of bond (*u*, *v*), **x**_*uvw*_ to be the feature of bond angle (*u*, *v*, *w*), and **x**_*uvw*x_ to be the feature of dihedral angle **x**_*uvw*_. We used the above three graphs, along with four initial features as inputs to our graph neural network E‐GeoGNN. The process of updating information is described in the Methods section.

### 2.2 Multiscale Self‐Supervised Learning Tasks

To further improve the ability of E‐GeoGNN to represent 3D geometric structures, we first designed four macro‐scale SSL tasks for E‐GeoGNN for 3D geometric structures: (1) predicting bond lengths, (2) predicting bond angles, (3) predicting dihedral angles, and (4) predicting the distances between two atoms. Among them, the prediction of bond lengths, bond angles, and dihedral angles is a local geometry prediction task, while the prediction of interatomic distances is a global geometry prediction task. In addition, to enable E‐GeoGNN to learn information about the electronic structure, we designed two additional SSL tasks that reflect the electronic structure of the molecule: (1) prediction of the CM5 charge of each atom in the molecule; (2) prediction of the Wiberg bond order of each chemical bond in the molecule.

#### 2.2.1 Self‐Supervised Learning of 3D Geometric Structure

In reference to GEM and other related studies, as illustrated in Figure 1b, we adopted a masking‐learning strategy to conduct local geometric SSL tasks aimed at predicting bond lengths, bond angles, and dihedral angles. First, we randomly selected 15% of the atoms in the molecule; for each selected atom, we extracted its first‐order neighborhood, including adjacent atoms and bonds, as well as the angles and dihedrals formed by the chosen atom. Subsequently, we masked the features of the selected atoms, bonds, angles, and dihedrals. Finally, we used the hidden states of the masked chemical bonds, angles, and dihedrals extracted using E‐GeoGNN after the last iteration to predict the corresponding bond lengths, bond angles, and dihedral angles, respectively. The methods for obtaining different conformations are in the Detail of Experimental Section, Supporting Information.

As shown in Figure 1b, for the global geometric task of predicting distances between pairs of atoms in a molecule, we constructed an atomic distance matrix based on the 3D coordinates of each molecule and subsequently predicted the distances between atom pairs. Notably, for two molecules with identical topological structures, the spatial distances between corresponding atoms may vary significantly. Therefore, for a given molecule, we did not treat the prediction of the atomic distance matrix as a regression task; we rather considered it as a multi‐class classification task by mapping the atomic distances into 30 equal‐width intervals. The task of predicting bond lengths can be viewed as a specific case of the atomic distance prediction task, with the former focusing more on local spatial structures, while the latter emphasizes the distribution of global spatial structures.

#### 2.2.2 Self‐Supervised Learning of Electronic Structure

Using the same strategy as that of the local geometric SSL task, we first randomly masked 15% of the atoms in the molecule along with their associated chemical bonds. Subsequently, we used the final hidden states to predict the CM5 charge of the masked atoms and the Wiberg bond order of the masked chemical bonds. Notably, unlike the pre‐training process for geometric structures, the molecular configuration inputs in the model in this phase are derived from high‐level theoretical calculations using DFT. In contrast, the configurations in the former process are obtained using a relatively coarse MMFF94 force field.^[^ ^43^ ^]^ The stability of these configurations enables the model to learn reliable information more effectively.^[^ ^44^ ^]^

Additionally, owing to the high cost of directly obtaining molecular quantum chemical properties through DFT calculations, we divided E‐GeoGNN pre‐training into two steps: (1) First, we conducted pre‐training at the 3D geometric level using a large‐scale unlabeled dataset, during which the molecular configurations were derived from MMFF94 force field calculations; 2) After completing the previous step, we performed two local electronic structure pre‐training tasks on our developed quantum chemical properties database, QuanDB. Through these two learning steps, we believe that E‐GeoGNN can effectively learn molecular structural information and demonstrate a strong ability to distinguish between the absolute configurations and electronic structural differences of molecules, enhancing the model's predictive performance. During E‐GeoGNN pre‐training, local and global 3D geometric structural information and electronic structural information of the molecules are considered, with the combined loss reflecting this integration. Details regarding the designed loss function are in the Methods section.

## 3 Experiments

To evaluate the predictive performance of Q‐GEM, we used MoleculeNet as a benchmark dataset and compared its model predictions with those of other baselines. Furthermore, we used QuanDB as an additional standard dataset to assess the capability of Q‐GEM in representing electronic structures.

### 3.1 Pre‐Training Settings

#### 3.1.1 Datasets

For the SSL of geometric and electronic structures, we used Zinc20 and QuanDB as the datasets for the two pre‐training stages. Zinc20 is a publicly available database comprising purchasable drug‐like compounds, from which we randomly selected 20,000,000 unlabeled molecules for this study. However, QuanDB is a recently developed quantum chemistry property database containing 154,610 distinct molecules, providing 53 global and 5 local quantum chemical properties, as well as one most stable conformation. This database offers a broader chemical space distribution than currently mainstream quantum chemistry property databases do.^[^ ^45^ ^]^ For both datasets, we randomly selected 90% of the samples for training, while the remaining 10% were used for model validation.

#### 3.1.2 Self‐Supervised Learning Task Settings

In the SSL of 3D geometric structures, we used RDKit to compute the conformations of 20 million molecules from the Zinc20 database under the MMFF94 force field as input for pre‐training. We derived the geometric features of the molecules, including bond lengths, bond angles, dihedral angles, and the distance matrix between atoms, from the obtained 3D coordinates. In the second phase of SSL focused on electronic structures, we used the lowest energy conformations from QuanDB as the input structures for E‐GeoGNN, with each atomic CM5 charge and the Wiberg bond order of chemical bonds serving as the pre‐training targets. Additionally, we explored pre‐training strategies using only 3D geometric structures and only electronic structures for subsequent experimental evaluations.

### 3.2 Molecular Property Prediction Settings

#### 3.2.1 Datasets

To facilitate comparisons with other studies, we used the MoleculeNet dataset as a benchmark for evaluating model predictive performance. The prediction encompassed classification (BACE, BBBP, ClinTox, SIDER, Tox21, ToxCast, HIV, and MUV) and regression (ESOL, Lipo, QM7, QM8, and QM9) tasks. Additionally, we used three types of atomic charges from QuanDB (NPA, ESPC, and Hirshfeld charge) to validate the model's ability to learn electronic structures. Following previous work,^[^ ^28^ ^]^ we applied a scaffold‐based partitioning method to all datasets, which segments molecules based on their carbon scaffolds. This approach ensures that the training and testing sets have similar chemical space distributions, providing a more accurate assessment of the model's generalization capabilities on imbalanced data samples.

#### 3.2.2 Evaluation Metrics

Following the recommendations from MoleculeNet, we used the average ROC‐AUC (area under the receiver operating characteristic curve) as the evaluation metric for classification tasks, which measures the performance of binary classification tasks (ranging from 0 to 1), with values closer to 1.0 indicating better model performance. For regression tasks, we used the root mean square error (RMSE) to evaluate ESOL and Lipo, while for QM7, QM8, and QM9, we used the mean absolute error (MAE). Both metrics have values ranging from 0 to 1, with values closer to 0 reflecting smaller prediction errors. Similarly, for the prediction tasks involving the three types of atomic charges from QuanDB, we applied RMSE to assess the predictive outcomes. For each prediction task, we conducted four independent predictions and subsequently calculated their mean and standard deviation.

#### 3.2.3 GNN Architecture

We defined the aggregation and combination functions using the Graph Isomorphism Network (GIN).^[^ ^46^ ^]^ To enhance performance further, we incorporated residual connections,^[^ ^47^ ^]^ layer normalization,^[^ ^48^ ^]^ and graph normalization.^[^ ^49^ ^]^ Finally, we used average pooling as the readout function to obtain the graph representation.

#### 3.2.4 Baselines

We compared Q‐GEM with various baseline GNN models. The non‐pretrained models included D‐MPNN,^[^ ^50^ ^]^ AttentiveFP,^[^ ^21^ ^]^ SGCN,^[^ ^51^ ^]^ DimeNet,^[^ ^52^ ^]^ and HMGNN,^[^ ^53^ ^]^ with SGCN, DimeNet, and HMGNN encoding 3D geometric structure information. The pre‐trained models included N‐Gram,^[^ ^54^ ^]^ PretrainGNN,^[^ ^23^ ^]^ GROVER,^[^ ^55^ ^]^ GraphMVP,^[^ ^34^ ^]^ and GEM.^[^ ^28^ ^]^ N‐Gram predicts molecular properties by combining node embeddings from short paths within the graph, utilizing random forests and extreme gradient boosting trees. PretrainGNN implements various SSL tasks, and we reported its best results as a comparison benchmark. GROVER integrates GNNs with Transformer^[^ ^56^ ^]^ and uses two SSL tasks, for which we reported experimental results at different network capacities, namely GROVER_base_ and GROVER_large_. GraphMVP represents the current state of molecular contrastive learning by introducing methods for 2D and 3D view contrastive learning. GEM inspired this study; however, its model exhibits limitations in representing dihedral angles and electronic structures.

## 4 Experimental Results and Discussion

### 4.1 Overall Performance

The overall performance of Q‐GEM in property prediction on MoleculeNet is presented in **Tables** **1** and **2**. Notably, Q‐GEM achieved state‐of‐the‐art (SOTA) results in 12 out of 13 downstream tasks. Compared with those of the previous SOTA model, GEM, Q‐GEM improved average performance in classification and regression tasks by 3.3% and 2.0%, respectively, across various datasets, while GEM achieved improvements of 8.8% and 4.7% over its predecessor. The limited gains from incorporating dihedral angles and electronic structure information into the prediction model, compared with GEM, suggest that the constraints of the benchmark dataset may play a significant role.^[^ ^57^ ^]^

**Table 1:** Performance of different models on 8 classification benchmarks. The mean and standard deviation of test ROC‐AUC (%) on 4 independent runs are reported.

	BACE	BBBP	ClinTox	SIDER	Tox21	ToxCast	HIV	MUV	Avg
D‐MPNN	0.809_(0.006)_	0.710_(0.003)_	^a)^0.906_(0.006)_	0.570_(0.007)_	0.759_(0.007)_	0.655_(0.003)_	0.771_(0.005)_	0.786_(0.014)_	0.746
AttentiveFP	0.784_(0.022)_	0.643_(0.018)_	0.847_(0.003)_	0.606_(0.032)_	0.761_(0.005)_	0.637_(0.002)_	0.757_(0.014)_	0.766_(0.015)_	0.726
N‐Gram_RF_	0.779_(0.015)_	0.697_(0.006)_	0.775_(0.040)_	0.668_(0.007)_	0.743_(0.004)_	n/a	0.772_(0.001)_	0.769_(0.007)_	n/a
N‐Gram_XGB_	0.791_(0.013)_	0.697_(0.006)_	0.875_(0.027)_	0.655_(0.007)_	0.758_(0.009)_	n/a	0.787_(0.004)_	0.748_(0.002)_	n/a
PretrainGNN	0.845(_0.007)_	0.687_(0.013)_	0.726_(0.015)_	0.627_(0.008)_	0.781_(0.006)_	0.657_(0.006)_	0.799_(0.007)_	0.813_(0.021)_	0.742
GraphMVP	0.812_(0.009)_	0.713_(0.016)_	0.803_ (0.018)_	0.639_(0.012)_	0.759_(0.005)_	0.631_(0.004)_	0.795_ (0.003)_	0.784_(0.012)_	0.742
GROVER_base_	0.826_(0.007)_	0.700_(0.001)_	0.812_(0.030)_	0.648_(0.006)_	0.743_(0.001)_	0.654_(0.004)_	0.625_(0.009)_	0.673_(0.018)_	0.710
GROVER_large_	0.810_(0.014)_	0.695_(0.001)_	0.762_(0.037)_	0.654_(0.001)_	0.735_(0.001)_	0.653_(0.005)_	0.682_(0.011)_	0.673_(0.018)_	0.708
GEM	**0.856**_(0.011)_	^a)^0.724_(0.004_ **_)_**	0.901_(0.013)_	^a)^0.672_(0.004)_	^a)^0.781_(0.001)_	**0.692**_(0.004)_	^a)^0.806_(0.009)_	**0 .817**_(0.005)_	^a)^0.781
Q‐GEM	**0 .856**_(0.008)_	**0.732**_(0.004)_	**0 .933**_(0.009)_	**0.673**_(0.008)_	**0.788**_(0.003)_	0.689_(0.002)_	**0 .840**_(0.008)_	**0 .817**_(0.020)_	**0 .791**

^a)^ These cells indicate the previous SOTA results.

**Table 2:** Performance of different models on 5 regression benchmarks. The mean and standard deviation of test RMSE and MAE on 4 independent runs are reported.

	RMSE (↓)		MAE (↓)		
	ESOL	Lipo	QM7	QM8	QM9
D‐MPNN	1.050_(0.008)_	0.683_(0.016)_	103.5_(8.6)_	0.0190_(0.0001)_	0.00814_(0.00001)_
AttentiveFP	0.877_(0.029)_	0.721_(0.001)_	72.0_(2.7)_	0.0179_(0.0001)_	0.00812_(0.00001)_
N‐Gram_RF_	1.074_(0.107)_	0.812_(0.028)_	92.8_(4.0)_	0.0236_(0.0006)_	0.01037_(0.00016)_
N‐Gram_XGB_	1.083_(0.082)_	2.072_(0.030)_	81.9_(1.9)_	0.0215_(0.0005)_	0.00964_(0.00031)_
PretrainGNN	1.100_(0.006)_	0.739_(0.003)_	113.2_(0.6)_	0.0200_(0.0001)_	0.00922_(0.00004)_
GROVER_base_	0.983_(0.090)_	0.817_(0.008)_	94.5_(3.8)_	0.0218_(0.0004)_	0.00984_(0.00055)_
GROVER_large_	0.895_(0.017)_	0.823_(0.010)_	92.0_(0.9)_	0.0224_(0.0003)_	0.00986_(0.00025)_
GEM	^a)^0.798_(0.029_ **_)_**	^a)^0.660_(0.008)_	^a)^58.9_(0.8)_	^a)^ **0.0171**_(0.0001)_	^a)^0.00746_(0.00001)_
Q‐GEM	**0.780**_(0.010)_	**0 .651**_(0.012)_	**55.3**_(0.3)_	**0.0171**_(0.0001)_	**0.00744**_(0.00008)_

^a)^ These cells indicate the previous SOTA results.

### 4.2 Contribution of Dihedral Angles

To assess the impact of incorporating dihedral angles on model prediction performance, we used a model without pre‐training it, specifically using E‐GeoGNN to extract molecular representations for regression tasks, as these endpoints are more susceptible to conformational effects than classification tasks. We compared various GNN architectures, including (1) commonly used GNN architectures, such as GIN, GAT, and GCN; (2) recent studies that incorporate 3D molecular geometry, including SGCN, DimeNet, HMGNN, and GeoGNN; (3) models specifically designed for molecular representation, such as D‐MPNN, AttentiveFP, and GTransformer. From **Table** **3**, we draw the following conclusions: (1) E‐GeoGNN achieved the best performance across the five regression tasks, reducing prediction error by 1.7% compared with that of the previous SOTA, GeoGNN, which only encodes bond lengths and angles, indicating that the inclusion of dihedral angle information enhances model prediction performance; (2) Additionally, on the QM8 and QM9 datasets, the performance of pre‐trained Q‐GEM showed minimal improvement over E‐GeoGNN, with MAE reductions of only 0.6% and 0.2%, respectively. This suggests that electronic structure SSL may not significantly contribute to the prediction of quantitative properties. This could be attributed to differences in the theoretical computational levels and chemical spaces between the QM datasets and the QuanDB used for our pre‐training. The specific differences are detailed in the Supporting Information.

**Table 3:** Performance of different GNN architectures for regression tasks. The mean and standard deviation of test RMSE and MAE on 4 independent runs are reported.

	RMSE (↓)		MAE (↓)		
	ESOL	Lipo	QM7	QM8	QM9
GIN	1.067_(0.051)_	0.757_(0.022)_	110.3_(7.2)_	0.0199_(0.0002)_	0.00886_(0.00005)_
GAT	1.556_(0.085)_	1.021_(0.029)_	103.0_(4.4)_	0.0224_(0.0005)_	0.01117_(0.00018)_
GCN	1.211_(0.052)_	0.773_(0.007)_	100.0_(3.8)_	0.0203_(0.0005)_	0.01117_(0.00018)_
D‐MPNN	1.050_(0.008)_	0.683_(0.016)_	103.5_(8.6)_	0.0190_(0.0001)_	0.00814_(0.00009)_
AttentiveFP	0.877_(0.029)_	0.721_(0.001)_	72.0_(2.7)_	0.0179_(0.0001)_	0.00812_(0.00001)_
GTransformer	2.298_(0.118)_	1.112_(0.029)_	161.3_(7.1)_	0.0361_(0.0008)_	0.00923_(0.00019)_
SGCN	1.629_(0.001)_	1.021_(0.013)_	131.3_(11.6)_	0.0285_(0.0005)_	0.01459_(0.00055)_
DimeNet	0.878_(0.023)_	0.727_(0.019)_	95.6_(4.1)_	0.0215_(0.0003)_	0.01031_(0.00076)_
HMGNN	1.390_(0.073)_	2.116_(0.473)_	101.6_(3.2)_	0.0249_(0.0004)_	0.01239_(0.0001)_
GeoGNN	0.832_(0.010)_	0.666_(0.015)_	59.0_(3.4)_	0.0173_(0.0004)_	0.00746_(0.00003)_
E‐GeoGNN	**0 .807**_(0.010)_	**0 .655**_(0.013)_	**57.2**_(0.6)_	**0 .0172**_(0.0001)_	**0 .00745**_(0.00003)_

^a)^ These cells indicate the previous SOTA results.

### 4.3 Contribution of the Electronic Structure

To assess the contribution of electronic SSL tasks to model prediction performance during pre‐training, we used various strategies to pretrain E‐GeoGNN using different SSL tasks and validated the model's performance on regression and classification datasets (**Tables** **4** and **5**). Q‐GEM_W.O_. indicates no pre‐training, Q‐GEM_GEO_ indicates SSL focused solely on 3D geometric structure, Q‐GEM_QCP_ indicates SSL focused only on electronic structure, and Q‐GEM represents the approach used in this study, which involved first conducting SSL for 3D geometric structure, followed by electronic structure SSL. The results indicated that 1) the pre‐training strategy used by Q‐GEM exhibits superior performance, with a 1.6% increase in ROC‐AUC for classification tasks compared with that of the no pre‐trained Q‐GEM_W.O._; 2) GEM_QCP_, with only 139 thousand training samples, achieved performance comparable to that of Q‐GEM_GEO_, which has 18 million training samples in classification tasks and outperformed Q‐GEM_GEO_ in all regression tasks. This suggests that incorporating electronic structure information aids model fitting, particularly in regression tasks that are more sensitive to conformational variations. Additionally, we discuss the contributions of different atomic charge types used in electronic structure SSL to model performance in the Tables S3 and S4, Supporting Information, where we demonstrated that the atomic charge type has a minimal impact on model performance.

**Table 4:** Performance of Q‐GEM with different pre‐training strategies for regression tasks. The mean and standard deviation of test RMSE and MAE on 4 independent runs are reported.

	RMSE (↓)		MAE (↓)		
	ESOL	Lipo	QM7	QM8	QM9
Q‐GEM_W.O._	0.807_(0.010)_	0.655_(0.013)_	57.2_(0.6)_	0.0172_(0.0001)_	0.00745_(0.00003)_
Q‐GEM_GEO_	0.794_(0.015)_	0.652_(0.004)_	57.0_(0.9)_	0.0173_(0.0002)_	0.00746_(0.00001)_
Q‐GEM_QCP_	0.788_(0.014)_	0.652_(0.015)_	56.4_(0.2)_	0.0172_(0.0002)_	0.00744_(0.00001)_
Q‐GEM	**0.780**_(0.010)_	**0 .651**_(0.012)_	**55.3**_(0.3)_	**0.0171**_(0.0001)_	**0.00744**_(0.00008)_

**Table 5:** Performance of Q‐GEM with 4 pre‐training strategies for classification tasks. The mean and standard deviation of test ROC‐AUC (%) on 4 independent runs are reported.

	BACE	BBBP	ClinTox	SIDER	Tox21	ToxCast	HIV	MUV	Avg.
Q‐GEM_W.O._	0.852_(0.008)_	0.729_(0.004)_	0.922_(0.003)_	0.653_(0.005)_	0.788_(0.004)_	0.684_(0.003)_	0.835_(0.007)_	0.751_(0.009)_	0.777
Q‐GEM_GEO_	0.842_(0.012)_	0.731_(0.008)_	**0 .938**_(0.016)_	0.664_(0.005)_	**0.789**_(0.002)_	0.685_(0.001)_	0.841_(0.011)_	0.800_(0.010)_	0.786
Q‐GEM_QCP_	0.854_(0.006)_	0.730_(0.009)_	0.920_(0.004)_	0.660_(0.005)_	0.784_(0.001)_	**0.687**_(0.004)_	**0 .843**_(0.002)_	**0 .817**_(0.009)_	0.787
Q‐GEM	**0 .856**_(0.008)_	**0.732**_(0.004)_	0.933_(0.009)_	**0.673**_(0.008)_	0.788_(0.003)_	0.689_(0.002)_	0.840_(0.008)_	**0 .817**_(0.020)_	**0 .791**

### 4.4 Ability to Distinguish Electronic structures

To validate the learning capability of Q‐GEM regarding electronic structures, we conducted model training and validation using the remaining 10% of the data from QuanDB, focusing on three types of atomic charges (NPA, ESPC, and Hirshfeld charge) as endpoints. We compared the results with those of different pre‐training strategies (**Table** **6**). The findings indicated that: (1) compared with the GEM model, Q‐GEM_GEO_ did not demonstrate an advantage in predicting local electronic structures, suggesting that merely adding information about dihedral angles is insufficient for the model to acquire the ability to represent electronic structure; (2) conversely, Q‐GEM_QCP_, which used only a few training samples, achieved a significant reduction in prediction error; (3) following pre‐training with two SSL levels, the prediction error was further decreased, indicating that SSL tasks targeting local electronic structures contribute to the model understanding of other electronic structure features of molecules, enhancing its ability to represent the electronic structure representation of the molecules.

**Table 6:** Performance of Q‐GEM for electronic structure learning. The mean and standard deviation of test MAE on 4 independent runs are reported.

	NPA	ESPC	Hirshfeld
GEM	0.0194_(0.0007)_	0.0965_(0.0005)_	0.0128_(0.00009)_
Q‐GEM_GEO_	0.0196_(0.0002)_	0.0963_(0.0006)_	0.0129_(0.00008)_
Q‐GEM_QCP_	0.0191_(0.0001)_	0.0947_(0.0007)_	**0.0124**_(0.00018)_
Q‐GEM	**0.0182**_(0.0003)_	**0.0945**_(0.0005)_	**0.0124**_(0.00028)_

### 4.5 Ability to Distinguish Stereoisomers

**Figure 2:**
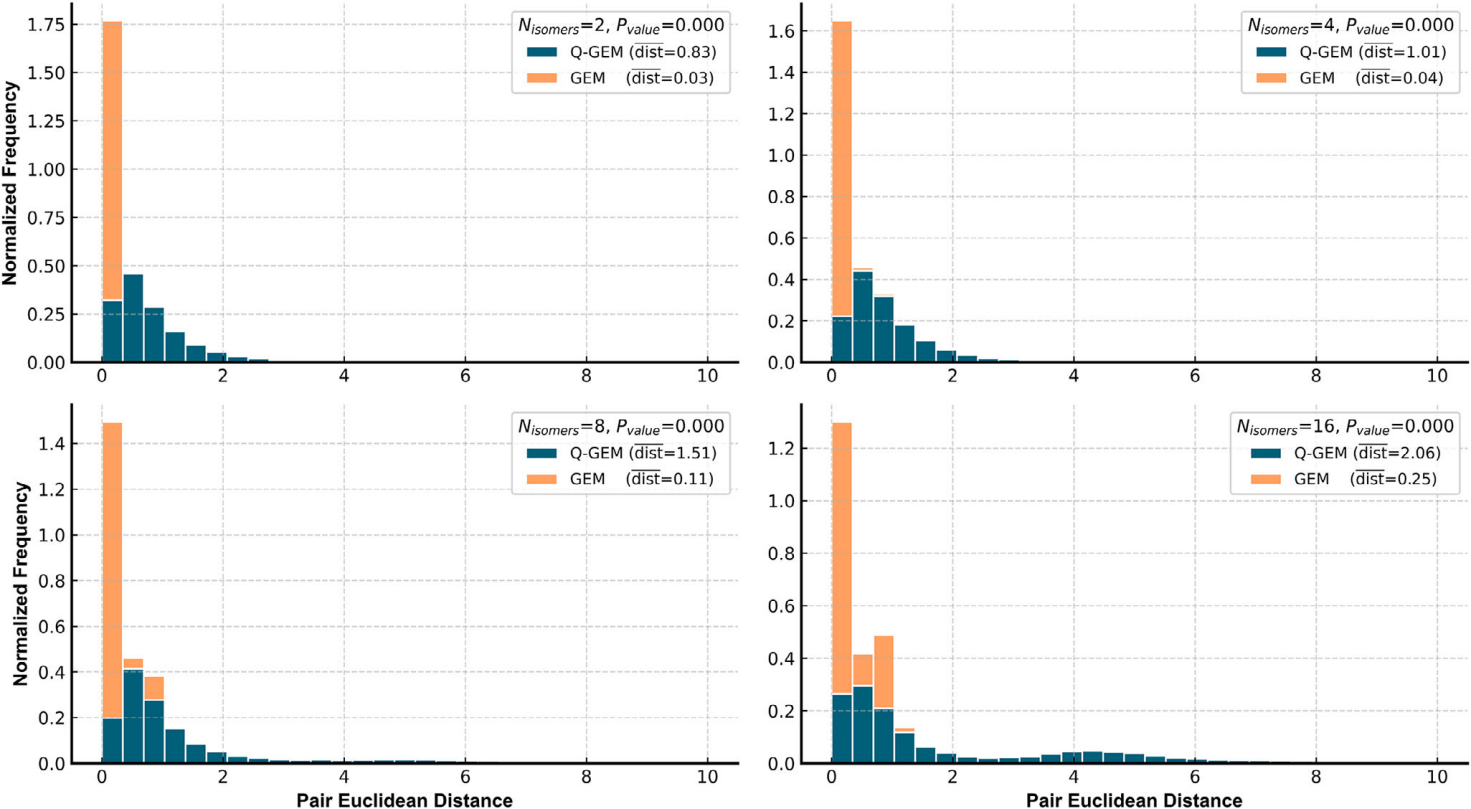
Euclidean distance of chirality set under different methods. The orange and blue colors represent GEM and Q‐GEM, respectively.

To assess the ability of Q‐GEM to differentiate chirality, we selected molecules with 2, 4, 8, and 16 chiral isomers from the ChemDiv database. We used RDKit to enumerate all isomers' Isomeric SMILES and optimized their conformations using the MMFF94 force field. We extracted the representations of these isomers using GEM and Q‐GEM_GEO_, followed by calculating the Euclidean distances between the representations of identical isomers. As illustrated in **Figure** **2**, the molecular representations extracted using GEM exhibited distances between enantiomers that were predominantly close to zero (with an average distance of 0.108). In contrast, the inclusion of dihedral angle information in Q‐GEM_GEO_ resulted in a substantial increase in the Euclidean distances between isomer representations (with an average distance of 5.410). This demonstrates that incorporating dihedral angle information significantly contributes to the model's ability to distinguish between different chirality of molecules.

**Figure 3:**
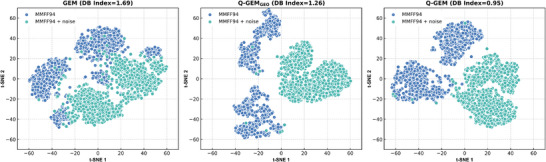
Pre‐trained representation visualization comparing different self‐supervised methods. Blue and green colors indicate the conformations obtained by MMFF94, and after applying noise in the former, respectively.

To further validate the impact of incorporating dihedral angles and electronic structure information on enhancing the ability to distinguish between molecular conformations, we randomly selected 1,000 molecules from ChemDiv. For each molecule, we first generated a reasonably accurate conformation using the MMFF94 force field. Subsequently, we introduced uniform noise distributed between 0 and 0.5 to the coordinates of each atom, creating a reference conformation (average RMSD, i.e., Root Mean Square Deviation, = 0.238). Next, we extracted features from both sets of conformations using GEM, Q‐GEM_GEO_, and Q‐GEM and visualized the output features in 2D space using the t‐SNE algorithm. Additionally, we calculated the Davies–Bouldin index to measure the degree of separation within the clusters, where a smaller Davies–Bouldin index indicates better clustering separation. As shown in **Figure** **3**, 1) Q‐GEM_GEO_, which incorporates dihedral angle information, effectively distinguished between the two conformations, with the Davies–Bouldin index decreasing from 1.69 for the GEM model to 1.26 for Q‐GEM_GEO_; 2) although the visual separation of features extracted by Q‐GEM_GEO_ was superior to that of Q‐GEM, its Davies–Bouldin index was higher than that of Q‐GEM (1.26 vs. 0.95). The conformations generated by MMFF94 were distinctly clustered into two groups, which increased the distance between samples with the same label, possibly owing to the neglect of the electronic structure similarity of the molecules.

### 4.6 Impact of the Quality of Molecular Conformers

As E‐GeoGNN requires encoding the bond lengths, bond angles, and dihedral angles of the molecular global structure, this can result in different molecular representations for various input conformations. To investigate the impact of different input conformations on molecular representation, we substituted the conformations originally computed by DFT with those obtained using the MMFF94 force field during the Q‐GEM_QCP_ phase. We explored enumerating 5 and 10 conformations, selecting the one with the lowest energy as input to the model. The prediction results for local charges with different input conformations are presented in **Table** **7**. Notably, models using DFT conformations displayed a slight superiority over those using MMFF94 conformations, achieving improvements of 0.9% and 0.5% for the 5 and 10 enumerated MMFF94 conformations, respectively. This indicates that high‐quality conformations enhance model performance significantly; however, the overall improvement remains relatively stable. However, considering the time cost associated with obtaining high‐precision conformations, such marginal performance gains may not be accepted. Consequently, the development of models for generating stable molecular conformations has increasingly become an emerging research focus,^[^ ^12^, ^58^, ^59^, ^60^ ^]^ with the potential to significantly reduce computational time while improving model performance.

**Table 7:** Performance of Q‐GEM for electronic structure learning. The mean and standard deviation of test MAE on 4 independent runs are reported.

	NPA	ESPC	Hirshfeld	Avg.
DFT	**0.0191**_(0.0001)_	**0.0947**_(0.0007)_	**0.0124**_(0.00018)_	**0.0421**
MMFF94_5	0.0195 _(0.0003)_	0.0950_(0.0003)_	0.0129_(0.00014)_	0.0425
MMFF94_10	0.0193_(0.0003)_	0.0949_(0.0007)_	0.0126_(0.00031)_	0.0423

## 5 Conclusion

To further explore the potential of molecular representation models, we proposed a novel property prediction framework, Q‐GEM. This framework comprises a 3D GNN for the comprehensive extraction of molecular 3D information, E‐GeoGNN, and SSL tasks at 3D geometric structures and electronic levels. Q‐GEM has achieved SOTA results in the majority of property prediction tasks. Additionally, we investigated the contributions of dihedral angles and electronic structural information to the model's performance, demonstrating that Q‐GEM possesses superior differentiation capabilities for electronic and geometric structures compared to other graph‐based molecular characterization models. This indicates that the model might have potential applications in predicting the properties of chiral molecules and addressing the issue of activity cliffs. It is important to note that this work does not account for long‐range atomic interactions within molecules or inter‐molecular interactions and only incorporates partial quantum chemical knowledge. In the future, we will expand the pre‐training dataset encompassing electronic and geometric structures and construct billion‐parameter models to develop a novel molecular representation large language model. This effort aims to strengthen model generalization capability and broaden its deployment across diverse molecular and material systems. Furthermore, integrating domain‐specific chemical knowledge rules during pre‐training is expected to enhance model interpretability while mitigating potential hallucination issues.

## 6 Methods

### 6.1 Information Update in E‐GeoGNN

E‐GeoGNN also functions as a message‐passing network. During the training process, the hidden states **h**_*uv*_ of bond and **h**_*uvw*_ of bond angle were used to communicate between graphs *G* and *H* and between graphs *H* and *I*. At the *t*‐th iteration: first, in the angle‐dihedral graph *I*, the information was aggregated from neighboring bond angles and the dihedral angles connecting them to update and obtain the new hidden state $h_{\mathrm{uvw}}^{t}$ of the bond angle:

(1) $$\begin{array}{ccc} a_{uvw}^{t} & = & \mathrm{Agg}._{I}^{t}\left(\left(\left(h_{uvw}^{t-1},h_{vwx}^{t-1},x_{uvwx}\right):x\in N\left(w\right)\right)\right.\\ & & \left.\cup \left(\left(h_{xuv}^{t-1},h_{uvw}^{t-1},x_{xuvw}\right):x\in N\left(u\right)\right)\right) \end{array}$$

(2) $$h_{uvw}^{t}=\mathrm{Comb}._{I}^{t}\left(h_{uvw}^{t-1},a_{uvw}^{t}\right)$$

Here, N(u) represents the atoms adjacent to atom *u*, and $(\left(u,v,w,x\right):x\in N\left(w\right)\cup \left(\left(x,u,v,w\right):x\in N\left(u\right)\right)$ represent the neighboring bond angles of the bond angle (*u*, *v*, *w*); $a_{uvw}^{t}$ is the information to be propagated, which was aggregated in the chemical bond‐bond angle graph *I* through the function Agg._*I*_ and updated from $h_{uvw}^{t-1}$ to $h_{uvw}^{t}$ through the function Comb._*I*_.

Subsequently, in the bond‐angle graph *H*, the information was aggregated from the neighboring nodes of the chemical bonds and the bond angles connecting them to obtain the hidden state $h_{uv}^{t}$ of the chemical bonds:

(3) $$\begin{array}{ccc} a_{uv}^{t} & = & \;\mathrm{Agg}._{H}^{t}\left(\left(\left(h_{uv}^{t-1},\;h_{vw}^{t-1},h_{uvw}^{t-1}\right):\;w\in N\left(v\right)\right)\right.\\ & & \left.\cup \left(\left(h_{wu}^{t-1},\;h_{uv}^{t-1},h_{wuv}^{t-1}\right):\;w\in N\left(u\right)\right)\right) \end{array}$$

(4) $$h_{uv}^{t}=\;\mathrm{Comb}._{H}^{t}\left(h_{uv}^{t-1},a_{uv}^{t}\right)$$

Here, $(\left(u,v,w\right):w\in N\left(v\right)\cup \left(\left(w,u,v\right):w\in N\left(u\right)\right)$ represents the neighboring bonds of (*u*, *v*); after aggregating the information to obtain $a_{uv}^{t}$ through the function Agg._*H*_, $h_{uv}^{t-1}$ was updated to $h_{uv}^{t}\;$ using the function Comb._*H*_.

Subsequently, in the atom‐bond graph *G*, similar to other GNNs, the information from the neighboring nodes of atoms and the chemical bonds connecting them was aggregated to obtain the current hidden state $h_{u}^{t}$ of the atom:

(5) $$a_{u}^{t}=\;\mathrm{Agg}._{G}^{t}\left(\left(\left(h_{u}^{t-1},h_{v}^{t-1},h_{uv}^{t-1}\right):v\in N\left(u\right)\right)\right)$$

(6) $$h_{u}^{t}=\;\mathrm{Comb}._{G}^{t}\left(h_{u}^{t-1},a_{u}^{t}\right)$$

Finally, the READOUT function was used to pool the set of hidden states of atoms obtained from the last iteration to obtain the feature vector **h**_*G*_ of the molecule:

(7) $$h_{G}=\mathrm{READOUT}(h_{u}^{K}\;|\;u\in V)$$

Here, *K* represents the number of iterations, **h**_*G*_ can be used for molecule‐level prediction tasks, while $h_{u}^{K}$ can be used for atom‐level prediction tasks. The input features of E‐GeoGNN can be found in Table S1, Supporting Information.

### 6.2 Loss Function of Multiscale SSL Tasks

For local 3D geometric structure SSL tasks, which involve predicting the bond length, bond angle, and dihedral angle of occluded targets, we have designed the following three loss functions:

(8) $$L_{length}\left(E\right)=\frac{1}{\left|E\right|}\sum_{\left(u,v\right)\in E}{\left(f_{length}\left(h_{u}^{K},h_{v}^{K}\right)-l_{uv}\right)}^{2}$$

(9) $$L_{angle}\;\left(A\right)=\frac{1}{\left|A\right|}\;\sum_{\left(u,v,w\right)\in A}{\left(f_{angle}\left(h_{u}^{K},h_{v}^{K},h_{w}^{K}\right)-\phi_{uvw}\right)}^{2}$$

(10) $$L_{dihedral}\left(D\right)=\frac{1}{\left|D\right|}\sum_{\left(u,v,w,x\right)\in D}{\left(f_{dihedral}\left(h_{u}^{K},h_{v}^{K},h_{w}^{K},h_{x}^{K}\right)-\theta_{uvw}\right)}^{2}$$

Here, *L_length_*, *L_angle_*, and *L_dihedral_* represent the loss functions in the prediction tasks of bond length, bond angle, and dihedral angle, respectively; *E*, *A*, and *D* represent the sets of bond, bond angle, and dihedral angle, respectively; *f_length_* (·), *f_angle_* (·), and *f_dihedral_* (·) represent the predicted values for bond length, bond angle, and dihedral angle, respectively; *l_uv_*, ϕ_*uvw*_, and θ_*uvw*_ represent the true values of bond length, bond angle, and dihedral angle, respectively.

The loss function for predicting the matrix of interatomic distances is defined as:

(11) $$L_{distance}\left(\nu \right)=\frac{1}{{\left|\nu \right|}^{2}}\sum_{u,v\in \nu }-\mathrm{bin}{\left(d_{uv}\right)}^{⊤}\cdot \log\left(f_{distance}\left(h_{u}^{K},h_{v}^{K}\right)\right)$$

Similarly, we have designed 2 loss functions for the 3D electronic structure SSL tasks:

(12) $$L_{NPA}\;\left(V\right)=\frac{1}{\left|u\right|}\;\sum_{u\in V}{\left(f_{NPA}\left(h_{u}^{K}\right)-\psi_{u}\right)}^{2}$$

(13) $$L_{Wiberg}\left(E\right)=\frac{1}{\left|E\right|}\sum_{\left(u,v\right)\in E}{\left(f_{Wiberg}\left(h_{u}^{K},h_{v}^{K}\right)-\omega_{uv}\right)}^{2}$$

Here, *f_NPA_* (·) and *f_Wiberg_* (·) represent the NPA charges and Wiberg bond orders predicted by the model, while ψ_*u*_ and ω_*uv*_ correspond to the charges and bond orders obtained through DFT calculations. The architecture of E‐GeoGNN and its SSL tasks are illustrated in Figure S1, Supporting Information

## Code Availability

The data and source code of this study is freely accessible at GitHub (https://github.com/kotori‐y/PaddleHelix‐GEM‐Improved/tree/gem‐advanced/apps/pretrained_compound/ChemRL/GEM) to allow replication of the results.

## Conflict of Interest

The authors declare no conflict of interest.

## Author Contributions

Z.Y. and L.W. are co‐first authors of this paper. Z.Y. was responsible for research design, manuscript writing, figure preparation, and core code development, while L.W. handled the overall experimental design, model construction, and contributed to manuscript writing and revisions. T.H. was responsible for code testing, and Y.W. and M.G. oversaw the visualization of experimental results. T.H., J.D., and J.X. are co‐corresponding authors of this paper. T.H. supervised quality control, provided conceptual guidance, and managed manuscript revisions; Junjie Ding offered support concerning computational resources and manuscript optimization; J.X., was responsible for the overall research concept, manuscript enhancement, and quality review.

## Supporting information



Supporting Information

## Data Availability

The data that support the findings of this study are openly available in [MolculeNet] at [https://moleculenet.org/], reference number [39].
