# Supplementary material for: Q‐GEM: Quantum Chemistry Knowledge Fusion Geometry‐Enhanced Molecular Representation for Property Prediction
Source: Adv Sci (Weinh). 2025 Jun 20;12(33):e04867. doi: 10.1002/advs.202504867 (PMC12412488; doi:10.1002/advs.202504867)
Supplement: Supplementary file 1 — Supporting Information [file ADVS-12-e04867-s001.docx]

# Supporting Information for: *Q-GEM: Quantum Chemistry Knowledge Fusion Geometry-Enhanced Molecular Representation for Property Prediction*

## Details for E-GeoGNN

### Model Architecture

As mentioned in the text, inspired by GEM, E-GeoGNN consists of three graph convolutional networks (GNN) blocks (Figure S1), namely the atom–bond graph, bond–angle graph, and angle–dihedral graph block. For each block, we continue to adopt graph isomorphism network (GIN)^[1]^ as the backbone of E-GeoGNN, and utilize common tricks in the graph neural network community such as Layer Norm,^[2]^ Graph Size Norm,^[3]^ and Residual Connection.^[4]^ In this study, the AGGREGATE and COMBINE functions of GIN are defined as follows:

$$\begin{aligned} \begin{aligned} {A\mathrm{GGRE}\mathrm{GATE}}_{angle-dihedral}: \mathbf{a}_{\mathrm{uvw}}^{t}=&\sum_{x\in\mathcal{N}\left( w \right)} \left( \mathbf{h}_{\mathrm{uvw}}^{t-1}+\mathbf{h}_{\mathrm{vwx}}^{t-1}+\mathbf{x}_{\mathrm{uvwx}} \right) \\ &+\sum_{x\in\mathcal{N}\left( u \right)} \left( \mathbf{h}_{\mathrm{uvw}}^{t-1}+\mathbf{h}_{\mathrm{xuv}}^{t-1}+\mathbf{x}_{\mathrm{xuvw}} \right) \end{aligned}\# \end{aligned}$$

$$\begin{aligned} {COMBINE}_{angle-dihedral}: \boldsymbol{h}_{uvw}^{t}=MLP\left( \boldsymbol{a}_{uvw}^{t} \right)\# \end{aligned}$$

$$\begin{aligned} \begin{aligned} {AGGREGATE}_{bond-angle}: \boldsymbol{a}_{uv}^{t}=&\sum_{w\in\mathcal{N}\left( v \right)} \left( \boldsymbol{h}_{uv}^{t-1}+\boldsymbol{h}_{vw}^{t-1}+\boldsymbol{x}_{uvw} \right) \\ &+\sum_{x\in\mathcal{N}\left( u \right)} \left( \boldsymbol{h}_{uv}^{t-1}+\boldsymbol{h}_{wv}^{t-1}+\boldsymbol{x}_{wuv} \right) \end{aligned}\# \end{aligned}$$

$$\begin{aligned} {COMBINE}_{bond-angle}: \boldsymbol{h}_{uv}^{t}=MLP\left( \boldsymbol{a}_{uv}^{t} \right)\# \end{aligned}$$

$$\begin{aligned} {AGGREGATE}_{atom-bond}: \boldsymbol{a}_{u}^{t}=\sum_{v\mathcal{\in N}\left( u \right)} \left( \boldsymbol{h}_{u}^{t-1}+\boldsymbol{h}_{v}^{t-1}+\boldsymbol{x}_{uv} \right)\# \end{aligned}$$

$$\begin{aligned} {COMBINE}_{atom-bond}: \boldsymbol{h}_{u}^{t}=MLP\left( \boldsymbol{a}_{u}^{t} \right)\# \end{aligned}$$

Where the $A\mathrm{GGRE}\mathrm{GATE}$ function summarizes the node features and the edge features, while $\mathrm{COMBINE}$ function is a 2-layer Multi Layer Perceptron (MLP) with hidden size of 32. We employ 8 GeoGNN blocks for atom-bond graph and bond-angle graph and the hidden size is set at 32.

As shown in Figure S1, based on E-GeoGNN, we devised self-supervised tasks at different scales. The self-supervised learning tasks related to three-dimensional geometric structures encompass predicting bond lengths, bond angles, dihedral angles, and interatomic distances, while the tasks related to electronic structure involve predicting atomic CM5 charges and Wiberg bond orders. More precisely, the headers are applied upon the node representations $\mathbf{h}_{u}^{K}$:

$$f_{length}\left( \text{h}_{u}^{K},\text{h}_{v}^{K} \right)=\text{MLP}\left( \text{Concat}\left( \text{h}_{u}^{K},\text{h}_{v}^{K} \right) \right)$$

$$f_{angle}\left( \text{h}_{u}^{K},\text{h}_{v}^{K},\text{h}_{w}^{K} \right)=\text{MLP}\left( \text{Concat}\left( \text{h}_{u}^{K},\text{h}_{v}^{K},\text{h}_{w}^{K} \right) \right)$$

$$f_{dihedral}\left( \text{h}_{u}^{K},\text{h}_{v}^{K},\text{h}_{w}^{K},\text{h}_{x}^{K} \right)=\text{MLP}\left( \text{Concat}\left( \text{h}_{u}^{K},\text{h}_{v}^{K},\text{h}_{w}^{K}, \text{h}_{x}^{K} \right) \right)$$

$$f_{distance}\left( \text{h}_{u}^{K},\text{h}_{v}^{K} \right)=\text{MLP}\left( \text{Concat}\left( \text{h}_{u}^{K},\text{h}_{v}^{K} \right) \right)$$

$$f_{cm5}\left( \text{h}_{u}^{K} \right)=\text{MLP}\left( \text{h}_{u}^{K} \right)$$

$$f_{wiberg}\left( \text{h}_{u}^{K},\text{h}_{v}^{K} \right)=\text{MLP}\left( \text{Concat}\left( \text{h}_{u}^{K},\text{h}_{v}^{K} \right) \right)$$

where $\mathrm{MLP}$ is a 2-layer MLP network with hidden size of 256 and $Concat$ is the concatenation operation. While for the graph-level tasks and downstream tasks, the headers are applied upon the graph representations $\mathbf{h}_{u}^{K}$:

$$f_{down_{x}}\left( \mathbf{h}_{G} \right)=\mathrm{MLP}(\mathbf{h}_{G})$$

where the $\mathrm{MLP}$ is a 3-layer $\mathrm{MLP}$ network with hidden size of 128.


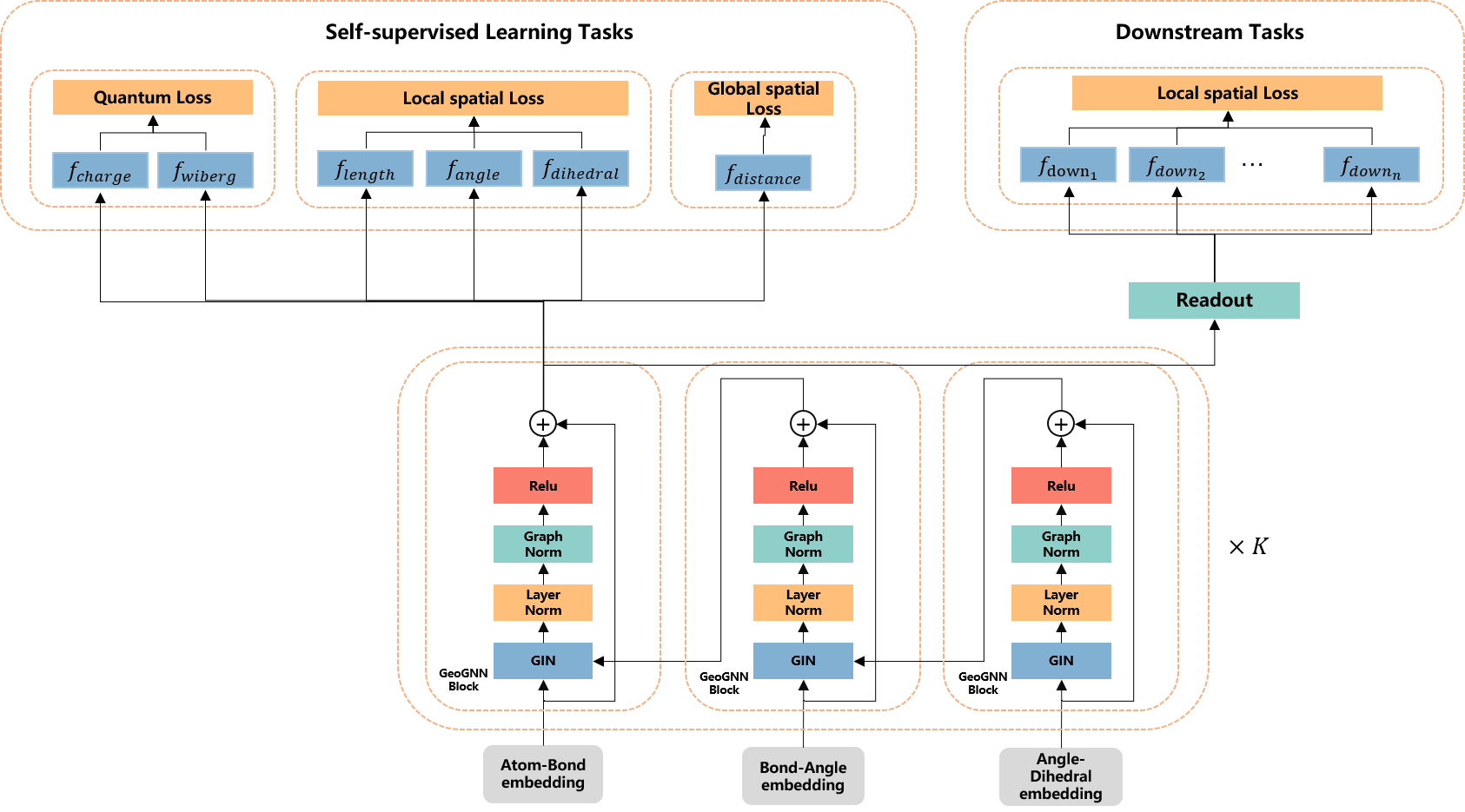


Figure S1 The architecture of E-GeoGNN and its SSL tasks

### Input Features

The input features for E-GeoGNN can be classified into four types: atomic features, bond features, bond angle features, and dihedral angle features (see Table S1). All features are computed by using RDKit. In the self-supervised learning tasks related to three-dimensional geometric structures, molecular conformations are calculated under the Merck molecular force field, while in the self-supervised learning tasks focusing on three-dimensional electronic structures, molecular conformations correspond to stable conformations obtained through DFT calculations. Among the input features, bond lengths, bond angles, and dihedral angles are continuous variables, while the remaining features are discrete variables. For the continuous variables, we employ Radial Basis Functions to convert each continuous value $x$ into an $M$-dimensional vector $e$:

$$e_{m}\left( x \right)=exp(-\gamma\left\| x-\mu_{m} \right\|^{2})$$

where γ controls the shape of the radial kernel, and we set γ = 10. $\left\{ \mu_{m} \right\}$is a list of centers ranging from the minimum value to the maximum value of corresponding features with stride of 0.1. Besides, for the discrete features, they are converted into one-hot vectors according to their vocabulary size.

Table S1 Input features of E-GeoGNN.

| **Feature type** | **Feature** | **Description** | **Size** |
| --- | --- | --- | --- |
| atom | atom type | type of atom (e.g., C, N, O), by atomic number (one-hot) | 119 |
|  | aromaticity | whether the atom is part of an aromatic system (one-hot) | 2 |
|  | formal charge | electrical charge (one-hot) | 16 |
|  | chirality tag | CW, CCW, unspecified or other(one-hot) | 4 |
|  | degree | number of covalent bonds (one-hot) | 11 |
|  | number of hydrogens | number of bonded hydrogen atoms (one-hot) | 9 |
|  | hybridization | sp, sp2, sp3, sp3d or sp3d2 (one-hot) | 5 |
| bond | bond dir | begin dash, begin wedge, etc. (one-hot) | 7 |
|  | bond type | single, double, triple or aromatic (one-hot) | 4 |
|  | in ring | whether the bond is part of a ring (one-hot) | 2 |
|  | bond length | bond length (float) | - |
| bond angle | bond angle | bond angle (float) | - |
| dihedral angle | dihedral angle | dihedral angle (float) | - |

## Details of Experimental Settings

### Computation of Rough Conformation

In the self-supervised learning (SSL) of 3D geometric structure, we utilized RDKit^[5]^ to obtain the conformations of the molecules, specifically employing the *AllChem.EmbedMolecule* function to generate initial three-dimensional conformations. During this process, RDKit takes the geometric constraints of the molecules into consideration and generates multiple potential conformations. After generating the initial conformations, we performed energy optimization using the *AllChem.MMFFOptimizeMolecule* method, which is based on the Merck Molecular Force Field. This approach effectively reduces the free energy of the molecules, yielding more stable conformations. Additionally, for each molecule, we employed the *AllChem.EmbedMultipleConformers* method to generate ten conformations, selecting the one with the lowest energy as the input conformation for the model. A comparison of QuanDB with other quantum chemistry property databases is presented in Table S2.

Table S2 Comparison of QuanDB with other quantum chemical property databases.

| **Datasets** | **Composition** | **Size** | **Method** | **Properties** | **Generated** |
| --- | --- | --- | --- | --- | --- |
| QM7 | C, O, N, S | 7,165 | PBE0 | electronic | Yes |
| QM9 | C, O, N, F | 0.13M | B3LYP/6-31G(2df,p) | electronic | Yes |
| QM8 | C, O, N, F | 0.2M | TDDFT, CC2/def2-TZVP | thermochemical | Yes |
| ANI-1 | C, O, N, F. | 20M | w97x/6-31G(D) | electronic | Yes |
| Alchemy | C, N, O, F, S, Cl | 0.12M | B3LYP/6-31G(2df,p) | electronic | Yes |
| QM7-X | C, N, O, S, Cl | 4.2M | PBE0+MBD. | electronic | Yes |
| QuanDB | C, N, O, P, S, F, Cl, Br | 0.16M | B3LYP-D3(BJ)/6-311G(d)/SMD/water  B3LYP-D3(BJ)/def2tzvp/SMD/water | electronic  thermochemical | Yes |

### DFT Computation of QuanDB

The calculation of the QC properties involves the following 3 steps: Firstly, he GMMX3.0 module in GaussView6.^[6]^ was used to search for molecular conformations. The obtained lowest energy conformation is then subjected to geometric structure optimization and frequency analysis using Gaussian16^[7]^ at the B3LYP-D3(BJ)/6-311G(d)/SMD/water theoretical level. After obtaining the lowest energy conformation without imaginary frequencies, the single-point energy calculation was performed at the B3LYP-D3(BJ)/def2-TZVP/SMD/water theoretical level. secondly, the Gaussian16 wavefunction file (.chk) was analyzed using Multiwfn software to obtain a .txt file containing the molecular electrostatic surface properties. Finally, QC properties were extracted automatically in batches using internal scripts. In total, we obtained 53 global and 5 local QC properties, as well as the lowest energy conformation for each molecule (Figure S1). Therefore, the QC properties in QuanDB are derived from three sources: (i) properties obtained from the geometric structure optimization and frequency analysis, (ii) properties calculated from the single-point energy of the lowest-energy conformation obtained in (i), and (ii) properties obtained from quantitative surface analysis of the wavefunction file using the Multiwfn software.^[8]^

### Pre-training and Downstream Fine-tuning

During the pre-training phase, we utilize parameters similar to those used in GEM, specifically employing distributed training with 8 GPUs and a batch size of 512 for each GPU. We set the dropout rate to 0.2, the vocabulary size for discretizing atomic distances to 30, and the mask ratio to 0.15. The Adam optimizer is used with a learning rate of 0.0002, and we train for 50 epochs for each pre-training method.

On the downstream fine-tuning stage, the molecules in each dataset are spitted into training set, validation set, and test set by the ratio of 8:1:1. We run each method for 100 epochs on the training set in the training process and then select the best epoch according to the validation set. The selected epoch is evaluated on the test set. We use Adam optimizer and train 100 epochs for each model. As the downstream tasks are sensitive to hyper-parameters, we apply a grid search on the dropout rate and the learning rate. For the dropout rate, we search $\left\{ 0.1,0.2,0.3,0.4 \right\}$.For the learning rate, we consider GeoGNN body and the downstream headers separately, where we search body-header learning rate pairs: $\left\{ \left( 0.001, 0.001 \right), \left( 0.001, 0.004 \right), \left( 0.004, 0.004 \right), \left( 0.0004, 0.004 \right),\left( 0.0001, 0.001 \right),\left( 0.0001, 0.0001 \right) \right\}$

### Visualization Details

For all the visualizations in the main text, we apply the same settings of the t-SNE algorithm to the graph representations $\text{h}_{G}$ for dimension reduction. More concretely, we firstly random sample 1,000 molecules for each cluster and apply PCA on their graph representations to reduce the dimension to be 50. Then t-SNE is applied to further reduce the dimension to be 2, with perplexity to be 20, maximal iteration to be 1,000, initial momentum to be 0.5, final momentum to be 0.8, and minimal gain to be 0.01. Finally, the 2-dimensional representations are plotted as 2D images.

## Additional Experiments

To determine the prediction targets used in the pre-training of electronic SSL, we explored various SSL targets in Q-GEM_QCP_, which focuses solely on electronic structure SSL. Specifically, we tested CM5, NPA, ESPC, and Hirshfeld charges as SSL objectives. As shown in Tables S3 and S4, the impact of using different charges as SSL targets on Q-GEM_QCP_ was minimal. Therefore, in this work, we adopted the prediction of smaller CM5 charges as the SSL target.

Table S3 Performance of Q-GEM_QCP_ with different charge target for regression tasks. The mean and standard deviation of test RMSE and MAE on 4 independent runs are reported.

|  | **RMSE (↓)** | | **MAE (↓)** | | |
| --- | --- | --- | --- | --- | --- |
|  | **ESOL** | **Lipo** | **QM7** | **QM8** | **QM9** |
| NPA | 0.798_(0.010)_ | 0.650_(0.010)_ | 56.5_(0.7)_ | 0.0172_(0.0001)_ | 0.00752_(0.00008)_ |
| ESPC | 0.792_(0.023)_ | 0.651_(0.007)_ | 56.4_(0.9)_ | 0.0172_(0.0002)_ | 0.00752_(0.00014)_ |
| Hirshfeld | 0.795_(0.011)_ | **0.647**_(0.007)_ | 56.5_(0.3)_ | **0.0171**_(0.0001)_ | **0.00740**_(0.00006)_ |
| CM5 | **0.780**_(0.010)_ | 0.651_(0.012)_ | 55.3_(0.3)_ | **0.0171**_(0.0001)_ | 0.00744_(0.00008)_ |

Table S4 Performance of Q-GEM_QCP_ with different charge target for classification tasks. The mean and standard deviation of test ROC-AUC (%) on 4 independent runs are reported.

|  | **BACE** | **BBBP** | **ClinTox** | **SIDER** | **Tox21** | **ToxCast** | **HIV** | **MUV** | **Avg.** |
| --- | --- | --- | --- | --- | --- | --- | --- | --- | --- |
| NPA | 0.853_(0.007)_ | 0.724_(0.012)_ | 0.920_(0.007)_ | 0.659_(0.007)_ | 0.788_(0.002)_ | 0.687_(0.004)_ | 0.840_(0.007)_ | 0.811_(0.035)_ | 0.785 |
| ESPC | 0.853_(0.007)_ | 0.723_(0.007)_ | **0.922**_(0.008)_ | 0.658_(0.005)_ | **0.789**_(0.002)_ | **0.689**_(0.001)_ | 0.839_(0.009)_ | 0.811_(0.011)_ | 0.786 |
| Hirshfeld | **0.856**_(0.020)_ | 0.728_(0.007)_ | 0.920_(0.003)_ | 0.654_(0.003)_ | 0.786_(0.005)_ | 0.684_(0.002)_ | **0.843**_(0.009)_ | 0.806_(0.005)_ | 0.785 |
| CM5 | 0.854_(0.006)_ | **0.730**_(0.009)_ | 0.920_(0.004)_ | **0.660_(_**_0.005)_ | 0.784_(0.001)_ | 0.687_(0.004)_ | **0.843**_(0.002)_ | **0.817**_(0.009)_ | 0.787 |

## Related Works

### Molecular Representation Methods

The current mainstream approach to molecular property prediction is the construction of quantitative structure-activity relationship models based on machine learning, which fundamentally assumes that structurally similar molecules possess similar physicochemical properties or biological activities.^[9]^ Therefore, molecular representation is still the key factor to carry out molecular property prediction.^[10-12]^ Traditional machine learning-driven property prediction models usually use molecular fingerprints to describe the 2D topological structures,^[13-15]^ such as extended-connectivity fingerprint (ECFP)^[16]^ and the molecular access system (MACCS)^[17]^ key. Additionally, some models compute inherent properties of molecules, such as molecular weight, atom count, lipophilicity, and the number of rings, to form molecular descriptors representing molecular structure.^[18]^ However, molecular fingerprints and descriptors require complex feature engineering, rely heavily on expert experience, and have limited ability to describe the molecular structure. Similarly, sequence models that directly consider the SMILES,^[19]^ International Union of Pure and Applied Chemistry Name,^[20]^ or self-referencing embedded strings (SELFIES)^[21]^ string structure of a molecule as input do not efficiently capture the geometric structural information of the molecule. Comparatively, the more chemically intuitive GNN method has become a current research hotspot in the field of molecular representation learning owing to its ability to directly extract molecular topological information.^[22-28]^

A molecular graph is denoted as $\mathcal{G=(}V, E)$, where $V$ represents the set of nodes, and $E$ represents the set of edges. In this representation, the atoms constituting the molecule are treated as nodes $v\in V$, while the edges connecting nodes $u$ and $v$ are denoted as $\left( u,v \right)\in E$. The GNN is a message propagation network,^[29]^ where the representation of node $v$ and the edge connecting this node to its neighboring node $u$ are denoted as $\mathbf{x}_{v}$and $\mathbf{x}_{uv}$, respectively. At a timestep, the GNN updates the current node by aggregating information from its neighboring nodes. After several iterations, the final hidden state for each atom (node), referred to as the atomic representation $\mathbf{h}_{v}$, can be used for various atom-level prediction tasks, such as metabolic site prediction.^[30]^ By applying pooling to $\mathbf{h}_{v}$, the molecular representation $\mathbf{h}_{G}$ can be obtained, which can be used for various molecular-level tasks, including property prediction, molecular generation, and conformation generation.

Since Duvenaud et al. first applied GNN for molecular representation in 2015,^[31]^ numerous GNN-based molecular representation methods have emerged.^[26-28]^ For instance, AttentiveFP effectively captures non-local features of graphs and interactions between distant nodes by considering long-range interactions among molecules;^[32]^ MGCN,^[33]^ based on graph convolutional networks, can capture multi-level quantum interactions from spatial information; and D-MPNN^[24]^ conducts edge-based message passing on directed graphs, simultaneously obtaining embeddings for atomic and chemical bond knowledge. Building on this, CoMPT^[34]^ employs a communication message passing model, improving the node-edge interaction kernel and applying the Transformer framework to capture long-range dependencies. MMGX^[35]^ achieves breakthroughs in both high-precision molecular property prediction and chemical interpretability by integrating hierarchical graph representations with self-attention mechanisms. MvMRL^[36]^ processes molecular graphs through a multi-scale GNN encoder while integrating SMILES, molecular graphs, and fingerprint features via dual cross-attention, generating comprehensive molecular representations that enhance prediction accuracy and robustness. However, despite the effectiveness of these methods in capturing various interatomic connections and interactions within molecules, they primarily focus on the 2D structural aspects of molecules and lack the capability to extract 3D geometric structural information.

### Geometric Deep Learning

As mentioned above, 2D GNNs only simply learn topological connectivity relationships in molecules and cannot accurately feature 3D structural information of molecules. However, integrating 3D coordinate information into the model enables the encoding of 3D geometrical structural properties of molecules, such as distances, angles, and dihedral angles, in Euclidean space. Therefore, 3D GNNs embedding molecular 3D structural information are receiving much attention in the field of accelerated drug and material discovery.^[37]^

Schütt et al. were pioneers in the field, and their construction of SchNet incorporated distance information in the information aggregation stage using continuous-filter convolutional layers,^[38]^ followed by DimeNet, which introduced information about angles into GNN for the first time to further enhance the representation of the model.^[39]^ However, these models only consider certain geometric parameters and overlook the significance of dihedral angles in molecular representation.^[25]^ SphereNet uses spherical coordinates to capture the 3D geometric structure information of molecules with invariance;^[25]^ however, there is no unique solution when Cartesian coordinates are transformed into spherical coordinates comprising distances, angles, and dihedral angles, and there is a certain degree of information loss. Moreover, SphereNet only guarantees the completeness of the local molecular representation and fails to consider how to transform the local into the completeness of the global representation.^[23]^ Wang et al. were the first to recognize this limitation and developed ComENet.^[23]^ ComENet attempts to encode the side chains of the entire molecule by fixing the rotational key between two rigid structures. By considering only one degree of freedom in the collinear plane, it fails to represent the molecule from a stereochemical perspective, resulting in a lack of uniqueness in the mapping from the molecular 3D structure to its final representation.^[40]^ In 2022, Fang et al. proposed a geometrically enhanced molecular representation learning method, GEM,^[41]^ upon which this study was built. Based on the traditional atom-bond graph, the method includes an additional bond-angle graph as input and strengthens the model to effectively differentiate between cis-trans isomerism by learning the global-scale bond lengths and angles, achieving good molecular property prediction. The model effectively distinguishes between cis and trans isomerism and achieves good molecular property prediction. GeminiMol^[42]^ introduces a GNN-based contrastive learning framework that simultaneously optimizes conformational space similarity and maximum common substructure similarity, enabling unified representations of molecular dynamic 3D conformations and 2D topological structures. Pre-GTM^[43]^, utilizing Gram matrix geometric representations combined with the Graphormer^[44]^ pre-training architecture, efficiently encodes 3D molecular conformational information and demonstrates improved performance in quantitative property prediction. 3DGCL^[45]^ employs a conformational pool and a 3D GNN encoder to effectively exploit molecular 3D structural information, using contrastive learning to further improve molecular property prediction. R-MAT^[46]^ incorporates radial basis functions and polynomial envelope functions within an enhanced relative self-attention mechanism, combining atomic distances with molecular graph topologies. This methodology dynamically models interatomic interactions, thereby enhancing the ability to capture complex chemical environments while addressing conventional monotonic distance constraints. Nevertheless, none of these methods systematically incorporates comprehensive dihedral angle information during the learning process.

Although many exploratory studies have been conducted on 3D GNN-based molecular representation, and significant progress has been achieved, we believe that a GNN model that completely extracts the global 3D geometrical structural information of a molecule remains lacking, possibly owing to the robust complexity caused by the introduction of dihedral angles, which consumes large computational cost. Hence, the existing methods are limited in their ability to distinguish the absolute molecule conformation that profoundly impacts molecular properties, which is possibly a significant bottleneck limiting the performance of molecular property prediction for further improvement. To address this challenge essentially, this study proposes a novel GNN model containing molecular "atom-bond length-bond angle-dihedral angle" information, which is used to extract the 3D geometric structure information of molecules and distinguishes the molecular absolute conformation through the clever design of targeted and effective training strategies.

### Pre-training for GNNs

Obtaining labeled data is challenging owing to the high cost of wet experiments, while collecting unlabeled data is easier. Therefore, many SSL methods have recently emerged, which have shown promising applications in molecular property prediction.^[20, 47, 48]^ Inspired by BERT model,^[49]^ a powerful pre-training model for natural language processing, SMILES-BERT utilizes the large-scale unlabeled SMILES dataset for masking learning of SMILES strings.^[19]^ However, since SMILES does not contain information about geometric structures, the application of pre-training strategies has gradually become significant in GNNs. SSL became common for pre-training of GNNs since PretrainGNN introduced the node-to-graph level SSL task in GNNs for the first time in 2019.^[50]^ AttrMask was the first attempt to apply, on a large-scale unlabeled dataset, the masked language modeling approach to assess molecular GNNs;^[51]^ subsequently, Mole-BERT was used to enhance its performance based on the BERT idea and accordingly, the first molecular graph-based BERT model was proposed.^[52]^ GEM and GraphMVP^[53]^ designed SSL tasks for 3D GNNs that contain molecular geometric information, which enables the models to capture some of the molecular geometric structural information. Recent studies have improved model performance and interpretability in downstream prediction tasks by integrating molecular substructures or domain-specific chemical knowledge as priors. For instance, SME^[54]^ utilizes the masking of chemically meaningful substructures to quantify their attribution scores based on prediction changes after masking. It further incorporates contrastive learning principles to guide structural optimization, thereby establishing a chemically intuitive explanatory framework. Meanwhile, RXGL^[55]^ models reactant-product relationships as latent vectors through a memory network, capturing reaction-level semantic correlations. It also designs a cross-view contrastive task to align representations from molecular graphs and reaction-aware graphs. This approach not only enhances performance in tasks such as product prediction and molecular property classification but also uncovers reaction-driven molecular relational patterns. HiMol^[56]^ integrates atom-level and graph-level SSL tasks: atom-level tasks reconstruct local molecular structures by predicting bond connectivity, atom types, and bond types, while graph-level tasks capture global properties by predicting the total number of atoms and bonds. TOML-BERT^[57]^ leverages pseudo-labeled data highly relevant to downstream tasks to learn domain-specific knowledge associated with target properties in advance. In addition, Automated3D^[58]^ introduces energy conformations as inputs to the model during the pre-training process, however, the conformational dataset used is limited by low conformational accuracy and a small molecular chemical space.^[59, 60]^ Furthermore, recent studies indicate that when the correlation between quantum mechanical descriptors and target properties is strong in small datasets, it can significantly enhance the predictive performance of models, particularly in the exploration of chemical reactivity predictions and the design of new drugs and materials.^[61]^ We briefly reviewed the most relevant studies here. The existing SSL methods primarily focus on the model's ability to learn geometric structural information, with no reported models specifically learning electronic structures closely related to molecular properties. This limitation stems primarily from the simplicity of the molecules in the common quantum chemistry property dataset, QM9,^[62]^ which contains molecules comprising at most nine heavy atoms and no more than 23 atoms in total, representing a relatively small chemical space, which is unsuitable for large-scale molecular pre-training. In this study, we designed an SSL task based on local quantum chemical properties, leveraging the latest quantum chemistry database,^[60]^ to equip the model with the capability to represent stable electronic structures. The comparison of different molecular representation methods is shown in Table S5.

Table S5 Comparison of different molecular representation methods.

| **Category** | **Model** | **3D Support** | **Angles** | **Dihedrals** | **Electronic Structure** | **Multimodal** | **Ref.** |
| --- | --- | --- | --- | --- | --- | --- | --- |
| Traditional | ECFP/MACCS | ✗ | ✗ | ✗ | ✗ | ✗ | ^[16, 17]^ |
| Sequence-based | SMILES-BERT | ✗ | ✗ | ✗ | ✗ | ✗ | ^[19]^ |
| 2D GNNs | AttentiveFP | ✗ | ✗ | ✗ | ✗ | ✗ | ^[32]^ |
|  | D-MPNN | ✗ | ✗ | ✗ | ✗ | ✗ | ^[24]^ |
|  | MvMRL | ✗ | ✗ | ✗ | ✗ | ✓ | ^[36]^ |
| 3D GNNs | SchNet | ✓ | ✗ | ✗ | ✗ | ✗ | ^[38]^ |
|  | DimeNet | ✓ | ✓ | ✗ | ✗ | ✗ | ^[39]^ |
|  | SphereNet | ✓ | ✓ | △ | ✗ | ✗ | ^[25]^ |
|  | ComENet | ✓ | ✓ | △ | ✗ | ✗ | ^[23]^ |
|  | 3DGCL | ✓ | ✓ | △ | ✗ | ✗ | ^[45]^ |
|  | GeminiMol | ✓ | ✓ | △ | ✗ | ✓ | ^[42]^ |
|  | Pre-GTM | ✓ | ✓ | △ | ✗ | ✗ | ^[43]^ |
|  | R-MAT | ✓ | ✓ | ✗ | ✗ | ✗ | ^[46]^ |
| Self-Supervised | MolCLR | ✓ | ✗ | ✗ | ✗ | ✗ | ^[63]^ |
|  | GEM | ✓ | ✓ | ✗ | ✗ | ✗ | ^[41]^ |
|  | GraphMVP | ✓ | ✓ | △ | ✗ | ✗ | ^[53]^ |
|  | SME | ✓ | ✓ | △ | ✗ | ✗ | ^[54]^ |
|  | HiMol | ✗ | ✗ | ✗ | ✗ | ✗ | ^[56]^ |
|  | TOML-BERT | ✗ | ✗ | ✗ | ✗ | ✗ | ^[57]^ |
|  | Q-GEM | ✓ | ✓ | ✓ | ✓ | ✗ | - |

✓ meant fully supports the feature; △ meant partially supports or with limitations; ✗ meant does not support

## Reference

[1] K. Xu, W. Hu, J. Leskovec, S. Jegelka, How Powerful are Graph Neural Networks?, International Conference on Learning Representations, 2018.

[2] J. Lei Ba, J.R. Kiros, G.E. Hinton, Layer Normalization, 2016, pp. arXiv:1607.06450.

[3] Y. Chen, X. Tang, X. Qi, C.-G. Li, R. Xiao, Learning Graph Normalization for Graph Neural Networks, arxiv preprint, 2020.

[4] K. He, X. Zhang, S. Ren, J. Sun, Deep Residual Learning for Image Recognition, 2016 IEEE Conference on Computer Vision and Pattern Recognition (CVPR), 2016, pp. 770-778.

[5] RDKit: Open-source cheminformatics., 2023.

[6] R. Dennington, T.A. Keith, J.M. Millam, GaussView Version 6.

[7] M.J. Frisch, G.W. Trucks, H.B. Schlegel, G.E. Scuseria, M.A. Robb, J.R. Cheeseman, G. Scalmani, V. Barone, G.A. Petersson, H. Nakatsuji, X. Li, M. Caricato, A.V. Marenich, J. Bloino, B.G. Janesko, R. Gomperts, B. Mennucci, H.P. Hratchian, J.V. Ortiz, A.F. Izmaylov, J.L. Sonnenberg, Williams, F. Ding, F. Lipparini, F. Egidi, J. Goings, B. Peng, A. Petrone, T. Henderson, D. Ranasinghe, V.G. Zakrzewski, J. Gao, N. Rega, G. Zheng, W. Liang, M. Hada, M. Ehara, K. Toyota, R. Fukuda, J. Hasegawa, M. Ishida, T. Nakajima, Y. Honda, O. Kitao, H. Nakai, T. Vreven, K. Throssell, J.A. Montgomery Jr., J.E. Peralta, F. Ogliaro, M.J. Bearpark, J.J. Heyd, E.N. Brothers, K.N. Kudin, V.N. Staroverov, T.A. Keith, R. Kobayashi, J. Normand, K. Raghavachari, A.P. Rendell, J.C. Burant, S.S. Iyengar, J. Tomasi, M. Cossi, J.M. Millam, M. Klene, C. Adamo, R. Cammi, J.W. Ochterski, R.L. Martin, K. Morokuma, O. Farkas, J.B. Foresman, D.J. Fox, Gaussian 16 Rev. C.01, Wallingford, CT, 2016.

[8] T. Lu, F. Chen, Multiwfn: a multifunctional wavefunction analyzer, J. Comput. Chem., 33 (2012) 580-592.

[9] C. Hansch, P.P. Maloney, T. Fujita, R.M. Muir, Correlation of Biological Activity of Phenoxyacetic Acids with Hammett Substituent Constants and Partition Coefficients, Nature, 194 (1962) 178-180.

[10] G.M. Crippen, T.F. Havel, Distance geometry and molecular conformation, Research Studies Press Taunton1988.

[11] R. Nilakantan, N. Bauman, J.S. Dixon, R. Venkataraghavan, Topological torsion: a new molecular descriptor for SAR applications. Comparison with other descriptors, J. Chem. Inf. Comput. Sci., 27 (1987) 82-85.

[12] R.E. Carhart, D.H. Smith, R. Venkataraghavan, Atom pairs as molecular features in structure-activity studies: definition and applications, J. Chem. Inf. Comput. Sci., 25 (1985) 64-73.

[13] L.L. Wang, J.J. Ding, L. Pan, L. Fu, J.H. Tian, D.S. Cao, H. Jiang, X.Q. Ding, Quantitative structure-toxicity relationship model for acute toxicity of organophosphates via multiple administration routes in rats and mice, J. Hazard. Mater., 401 (2021) 123724.

[14] N.N. Wang, J. Dong, Y.H. Deng, M.F. Zhu, M. Wen, Z.J. Yao, A.P. Lu, J.B. Wang, D.S. Cao, ADME Properties Evaluation in Drug Discovery: Prediction of Caco-2 Cell Permeability Using a Combination of NSGA-II and Boosting, J. Chem. Inf. Model., 56 (2016) 763-773.

[15] J. Dong, N.N. Wang, Z.J. Yao, L. Zhang, Y. Cheng, D. Ouyang, A.P. Lu, D.S. Cao, ADMETlab: a platform for systematic ADMET evaluation based on a comprehensively collected ADMET database, Journal of cheminformatics, 10 (2018) 29.

[16] D. Rogers, M. Hahn, Extended-Connectivity Fingerprints, J. Chem. Inf. Model., 50 (2010) 742-754.

[17] J.L. Durant, B.A. Leland, D.R. Henry, J.G. Nourse, Reoptimization of MDL Keys for Use in Drug Discovery, J. Chem. Inf. Comput. Sci., 42 (2002) 1273-1280.

[18] Z.Y. Yang, Z.J. Yang, A.P. Lu, T.J. Hou, D.S. Cao, Scopy: an integrated negative design python library for desirable HTS/VS database design, Brief. Bioinform., 22 (2021).

[19] S. Wang, Y. Guo, Y. Wang, H. Sun, J. Huang, Smiles-bert: large scale unsupervised pre-training for molecular property prediction, Proceedings of the 10th ACM international conference on bioinformatics, computational biology and health informatics, 2019, pp. 429-436.

[20] J. Mao, J. Wang, A. Zeb, K.-H. Cho, H. Jin, J. Kim, O. Lee, Y. Wang, K.T. No, Transformer-Based Molecular Generative Model for Antiviral Drug Design, J. Chem. Inf. Model., 64 (2024) 2733-2745.

[21] M. Krenn, F. Häse, A. Nigam, P. Friederich, A. Aspuru-Guzik, Self-referencing embedded strings (SELFIES): A 100% robust molecular string representation, Machine Learning: Science and Technology, 1 (2020) 045024.

[22] R. Miao, D. Liu, L. Mao, X. Chen, L. Zhang, Z. Yuan, S. Shi, H. Li, S. Li, GR-pKa: a message-passing neural network with retention mechanism for pKa prediction, Brief. Bioinform., 25 (2024).

[23] L. Wang, Y. Liu, Y.-C. Lin, H. Liu, S. Ji, ComENet: Towards Complete and Efficient Message Passing for 3D Molecular Graphs, arxiv preprint, 2022.

[24] X. Han, M. Jia, Y. Chang, Y. Li, S. Wu, Directed message passing neural network (D-MPNN) with graph edge attention (GEA) for property prediction of biofuel-relevant species, Energy and AI, 10 (2022) 100201.

[25] Y. Liu, L. Wang, M. Liu, X. Zhang, B. Oztekin, S. Ji, Spherical Message Passing for 3D Molecular Graphs, arxiv preprint, 2021.

[26] S. Kearnes, K. McCloskey, M. Berndl, V. Pande, P. Riley, Molecular graph convolutions: moving beyond fingerprints, J. Comput-Aided. Mol. Des., 30 (2016) 595-608.

[27] C. Chen, W. Ye, Y. Zuo, C. Zheng, S.P. Ong, Graph Networks as a Universal Machine Learning Framework for Molecules and Crystals, Chem. Mater., 31 (2019) 3564-3572.

[28] K. Yang, K. Swanson, W. Jin, C. Coley, P. Eiden, H. Gao, A. Guzman-Perez, T. Hopper, B. Kelley, M. Mathea, A. Palmer, V. Settels, T. Jaakkola, K. Jensen, R. Barzilay, Analyzing Learned Molecular Representations for Property Prediction, J. Chem. Inf. Model., 59 (2019) 3370-3388.

[29] J. Gilmer, S.S. Schoenholz, P.F. Riley, O. Vinyals, G.E. Dahl, Neural message passing for quantum chemistry, arxiv preprint, 2017.

[30] D. Jiang, Z. Ye, C.-Y. Hsieh, Z. Yang, X. Zhang, Y. Kang, H. Du, Z. Wu, J. Wang, Y. Zeng, H. Zhang, X. Wang, M. Wang, X. Yao, S. Zhang, J. Wu, T. Hou, MetalProGNet: a structure-based deep graph model for metalloprotein–ligand interaction predictions, Chem. Sci., 14 (2023) 2054-2069.

[31] D.K. Duvenaud, D. Maclaurin, J. Iparraguirre, R. Bombarell, T. Hirzel, A. Aspuru-Guzik, R.P. Adams, Convolutional networks on graphs for learning molecular fingerprints, arxiv preprint, 2015.

[32] Z. Xiong, D. Wang, X. Liu, F. Zhong, X. Wan, X. Li, Z. Li, X. Luo, K. Chen, H. Jiang, M. Zheng, Pushing the Boundaries of Molecular Representation for Drug Discovery with the Graph Attention Mechanism, J. Med. Chem., 63 (2020) 8749-8760.

[33] C. Lu, Q. Liu, C. Wang, Z. Huang, P. Lin, L. He, Molecular Property Prediction: A Multilevel Quantum Interactions Modeling Perspective, Proceedings of the Thirty-Third AAAI Conference on Artificial Intelligence and Thirty-First Innovative Applications of Artificial Intelligence Conference and Ninth AAAI Symposium on Educational Advances in Artificial Intelligence, 2019, pp. Article 130.

[34] J. Chen, S. Zheng, Y. Song, J. Rao, Y. Yang, Learning Attributed Graph Representations with Communicative Message Passing Transformer, International Joint Conference on Artificial Intelligence, 2021.

[35] A. Kengkanna, M. Ohue, Enhancing property and activity prediction and interpretation using multiple molecular graph representations with MMGX, Communications Chemistry, 7 (2024) 74.

[36] R. Zhang, Y. Lin, Y. Wu, L. Deng, H. Zhang, M. Liao, Y. Peng, MvMRL: a multi-view molecular representation learning method for molecular property prediction, Brief. Bioinform., 25 (2024).

[37] K. Atz, F. Grisoni, G. Schneider, Geometric deep learning on molecular representations, Nat. Mach. Intell., 3 (2021) 1023-1032.

[38] K.T. Schütt, P.-J. Kindermans, H.E. Sauceda, S. Chmiela, A. Tkatchenko, K.-R. Müller, SchNet: a continuous-filter convolutional neural network for modeling quantum interactions, Proceedings of the 31st International Conference on Neural Information Processing Systems, Curran Associates Inc., Long Beach, California, USA, 2017, pp. 992–1002.

[39] J. Klicpera, J. Groß, S. Günnemann, Directional message passing for molecular graphs, arxiv preprint, 2020.

[40] W. Du, Y. Du, L. Wang, D. Feng, G. Wang, S. Ji, C.P. Gomes, Z. Ma, A new perspective on building efficient and expressive 3D equivariant graph neural networks, arxiv preprint, 2023.

[41] X. Fang, L. Liu, J. Lei, D. He, S. Zhang, J. Zhou, F. Wang, H. Wu, H. Wang, Geometry-enhanced molecular representation learning for property prediction, Nat. Mach. Intell., 4 (2022) 127-134.

[42] L. Wang, S. Wang, H. Yang, S. Li, X. Wang, Y. Zhou, S. Tian, L. Liu, F. Bai, Conformational Space Profiling Enhances Generic Molecular Representation for AI-Powered Ligand-Based Drug Discovery, Advanced Science, 11 (2024) 2403998.

[43] W. Xiang, F. Zhong, L. Ni, M. Zheng, X. Li, Q. Shi, D. Wang, Gram matrix: an efficient representation of molecular conformation and learning objective for molecular pretraining, Brief. Bioinform., 25 (2024).

[44] C. Ying, T. Cai, S. Luo, S. Zheng, G. Ke, D. He, Y. Shen, T.-Y. Liu, Do Transformers Really Perform Bad for Graph Representation?, 2021, pp. arXiv:2106.05234.

[45] K. Moon, H.-J. Im, S. Kwon, 3D graph contrastive learning for molecular property prediction, Bioinformatics, 39 (2023).

[46] Ł. Maziarka, D. Majchrowski, T. Danel, P. Gaiński, J. Tabor, I. Podolak, P. Morkisz, S. Jastrzębski, Relative molecule self-attention transformer, Journal of cheminformatics, 16 (2024) 3.

[47] Y. Ni, S. Feng, X. Hong, Y. Sun, W.-Y. Ma, Z.-M. Ma, Q. Ye, Y. Lan, Pre-training with fractional denoising to enhance molecular property prediction, Nat. Mach. Intell., 6 (2024) 1169-1178.

[48] D. Buterez, J.P. Janet, S.J. Kiddle, D. Oglic, P. Lió, Transfer learning with graph neural networks for improved molecular property prediction in the multi-fidelity setting, Nat. Commun., 15 (2024) 1517.

[49] J. Devlin, M.-W. Chang, K. Lee, K. Toutanova, Bert: Pre-training of deep bidirectional transformers for language understanding, arxiv preprint, 2019.

[50] W. Hu, B. Liu, J. Gomes, M. Zitnik, P. Liang, V. Pande, J. Leskovec, Strategies for Pre-training Graph Neural Networks, arxiv preprint, 2019.

[51] Z. Hu, Y. Dong, K. Wang, K.-W. Chang, Y. Sun, GPT-GNN: Generative Pre-Training of Graph Neural Networks, Proceedings of the 26th ACM SIGKDD International Conference on Knowledge Discovery & Data Mining, 2020.

[52] J. Xia, C. Zhao, B. Hu, Z. Gao, C. Tan, Y. Liu, S. Li, S.Z. Li, Mole-BERT: Rethinking Pre-training Graph Neural Networks for Molecules, The Eleventh International Conference on Learning Representations, 2023.

[53] S. Liu, H. Wang, W. Liu, J. Lasenby, H. Guo, J. Tang, Pre-training Molecular Graph Representation with 3D Geometry, International Conference on Learning Representations, 2022.

[54] Z. Wu, J. Wang, H. Du, D. Jiang, Y. Kang, D. Li, P. Pan, Y. Deng, D. Cao, C.-Y. Hsieh, T. Hou, Chemistry-intuitive explanation of graph neural networks for molecular property prediction with substructure masking, Nat. Commun., 14 (2023) 2585.

[55] A. Li, E. Casiraghi, J. Rousu, Chemical reaction enhanced graph learning for molecule representation, Bioinformatics, 40 (2024).

[56] X. Zang, X. Zhao, B. Tang, Hierarchical Molecular Graph Self-Supervised Learning for property prediction, Communications Chemistry, 6 (2023) 34.

[57] Y. Duan, X. Yang, X. Zeng, W. Wang, Y. Deng, D. Cao, Enhancing Molecular Property Prediction through Task-Oriented Transfer Learning: Integrating Universal Structural Insights and Domain-Specific Knowledge, J. Med. Chem., 67 (2024) 9575-9586.

[58] X. Wang, H. Zhao, W. Tu, Q. Yao, Automated 3D Pre-Training for Molecular Property Prediction, arxiv preprint, 2023.

[59] W. Hu, M. Fey, H. Ren, M. Nakata, Y. Dong, J. Leskovec, OGB-LSC: A Large-Scale Challenge for Machine Learning on Graphs, arxiv preprint, 2021.

[60] Z. Yang, T. Huang, L. Pan, J. Wang, L. Wang, J. Ding, J. Xiao, QuanDB: a quantum chemical property database towards enhancing 3D molecular representation learning, Journal of cheminformatics, 16 (2024) 48.

[61] S.-C. Li, H. Wu, A. Menon, K.A. Spiekermann, Y.-P. Li, W.H. Green, When Do Quantum Mechanical Descriptors Help Graph Neural Networks to Predict Chemical Properties?, J. Am. Chem. Soc., 146 (2024) 23103-23120.

[62] R. Ramakrishnan, P.O. Dral, M. Rupp, O.A. von Lilienfeld, Quantum chemistry structures and properties of 134 kilo molecules, Sci. Data, 1 (2014) 140022.

[63] Y. Wang, J. Wang, Z. Cao, A. Barati Farimani, Molecular contrastive learning of representations via graph neural networks, Nat. Mach. Intell., 4 (2022) 279-287.
